# Sex differences in dementia risk and risk factors: Individual‐participant data analysis using 21 cohorts across six continents from the COSMIC consortium

**DOI:** 10.1002/alz.12962

**Published:** 2023-02-15

**Authors:** Jessica Gong, Katie Harris, Darren M. Lipnicki, Erico Castro‐Costa, Maria Fernanda Lima‐Costa, Breno S. Diniz, Shifu Xiao, Richard B. Lipton, Mindy J. Katz, Cuiling Wang, Pierre‐Marie Preux, Maëlenn Guerchet, Antoine Gbessemehlan, Karen Ritchie, Marie‐Laure Ancelin, Ingmar Skoog, Jenna Najar, Therese Rydberg Sterner, Nikolaos Scarmeas, Mary Yannakoulia, Mary H. Kosmidis, Antonio Guaita, Elena Rolandi, Annalisa Davin, Oye Gureje, Stella Trompet, Jacobijn Gussekloo, Steffi Riedel‐Heller, Alexander Pabst, Susanne Röhr, Suzana Shahar, Devinder Kaur Ajit Singh, Nurul Fatin Malek Rivan, Martin van Boxtel, Sebastian Köhler, Mary Ganguli, Chung‐Chou Chang, Erin Jacobsen, Mary Haan, Ding Ding, Qianhua Zhao, Zhenxu Xiao, Kenji Narazaki, Tao Chen, Sanmei Chen, Tze Pin Ng, Xinyi Gwee, Katya Numbers, Karen A. Mather, Marcia Scazufca, Antonio Lobo, Concepción De‐la‐Cámara, Elena Lobo, Perminder S. Sachdev, Henry Brodaty, Maree L. Hackett, Sanne A. E. Peters, Mark Woodward, for the Cohort Studies of Memory in an International Consortium (COSMIC)

**Affiliations:** ^1^ The George Institute for Global Health University of New South Wales Sydney Australia; ^2^ The George Institute for Global Health Imperial College London London UK; ^3^ Centre for Healthy Brain Ageing (CHeBA) Discipline of Psychiatry and Mental Health Faculty of Medicine and Health UNSW Sydney Sydney Australia; ^4^ Center for Studies in Public Health and Aging Rene Rachou Institute Oswaldo Cruz Foundation Belo Horizonte Brazil; ^5^ UConn Center on Aging Department of Psychiatry School of Medicine University of Connecticut Health Center Farmington Connecticut USA; ^6^ Department of Geriatric Psychiatry Shanghai Mental Health Centre Shanghai Jiaotong University School of Medicine Shanghai China; ^7^ Department of Neurology Albert Einstein College of Medicine Bronx New York USA; ^8^ Department of Epidemiology and Community Heath Albert Einstein College of Medicine Bronx New York USA; ^9^ Inserm U1094, IRD U270, Univ. Limoges CHU Limoges, EpiMaCT ‐ Epidemiology of chronic diseases in tropical zone Institute of Epidemiology and Tropical Neurology OmegaHealth Limoges France; ^10^ INM Institute for Neurosciences of Montpellier Univ Montpellier INSERM Montpellier France; ^11^ Department of Psychiatry and Neurochemistry Center for Ageing and Health (Age Cap) University of Gothenburg Gothenburg Sweden; ^12^ 1st Department of Neurology Aiginition Hospital National and Kapodistrian University of Athens Medical School Athens Greece; ^13^ Department of Neurology Columbia University New York New York USA; ^14^ Department of Nutrition and Dietetics Harokopio University Athens Greece; ^15^ Lab of Cognitive Neuroscience School of Psychology Aristotle University of Thessaloniki Thessaloniki Greece; ^16^ Golgi Cenci Foundation Abbiategrasso Italy; ^17^ Department of Brain and Behavioral Sciences University of Pavia Pavia Italy; ^18^ WHO Collaborating Centre for Research and Training in Mental Health Neurosciences and Substance Abuse Department of Psychiatry University of Ibadan Ibadan Nigeria; ^19^ Section of Gerontology and Geriatrics Department of Internal Medicine Leiden University Medical Center Leiden the Netherlands; ^20^ Department of Public Health and Primary Care Leiden the Netherlands; ^21^ Institute of Social Medicine Occupational Health and Public Health (ISAP) University of Leipzig Leipzig Germany; ^22^ Centre for Healthy Ageing and Wellness Universiti Kebangsaan Malaysia Kuala Lumpur Malaysia; ^23^ Alzheimer Centrum Limburg School for Mental Health and Neuroscience Maastricht University Maastricht the Netherlands; ^24^ Department of Medicine University of Pittsburgh Pittsburgh Pennsylvania USA; ^25^ Department of Epidemiology and Biostatistics School of Medicine University of California, San Francisco San Francisco California USA; ^26^ Institute of Neurology National Center for Neurological Disorders National Clinical Research Center for Aging and Medicine Huashan Hospital Fudan University Shanghai China; ^27^ Center for Liberal Arts Fukuoka Institute of Technology Fukuoka Japan; ^28^ Sports and Health Research Center Department of Physical Education Tongji University Shanghai China; ^29^ Global Health Nursing Department of Health Sciences Graduate School of Biomedical and Health Sciences Hiroshima University Hiroshima Japan; ^30^ Gerontology Research Programme Department of Psychological Medicine Yong Loo Lin School of Medicine National University of Singapore Queenstown Singapore; ^31^ Instituto de Psiquiátria e LIM‐23 Hospital da Clínicas Faculdade de Medicina Universidade de São Paulo São Paulo Brazil; ^32^ Department of Medicine and Psychiatry Universidad de Zaragoza Zaragoza Spain; ^33^ Instituto de Investigación Sanitaria Aragón (IIS Aragón) Zaragoza Spain; ^34^ n°33 CIBERSAM Madrid Spain; ^35^ Department of Public Health Universidad de Zaragoza Zaragoza Spain; ^36^ Faculty of Health and Wellbeing University of Central Lancashire Lancashire UK; ^37^ Julius Center for Health Sciences and Primary Care University Medical Center Utrecht Utrecht University Utrecht the Netherlands

**Keywords:** data harmonization, dementia, diversity, risk factor, sex difference

## Abstract

**Introduction:**

Sex differences in dementia risk, and risk factor (RF) associations with dementia, remain uncertain across diverse ethno‐regional groups.

**Methods:**

A total of 29,850 participants (58% women) from 21 cohorts across six continents were included in an individual participant data meta‐analysis. Sex‐specific hazard ratios (HRs), and women‐to‐men ratio of hazard ratios (RHRs) for associations between RFs and all‐cause dementia were derived from mixed‐effect Cox models.

**Results:**

Incident dementia occurred in 2089 (66% women) participants over 4.6 years (median). Women had higher dementia risk (HR, 1.12 [1.02, 1.23]) than men, particularly in low‐ and lower‐middle‐income economies. Associations between longer education and former alcohol use with dementia risk (RHR, 1.01 [1.00, 1.03] per year, and 0.55 [0.38, 0.79], respectively) were stronger for men than women; otherwise, there were no discernible sex differences in other RFs.

**Discussion:**

Dementia risk was higher in women than men, with possible variations by country‐level income settings, but most RFs appear to work similarly in women and men.

## 1 INTRODUCTION

The number of people living with dementia is projected to exceed 150 million by 2050 worldwide, three times the 50 million estimated in 2019.^1^ There are strong social and economic imperatives to address the increasing burden associated with dementia, particularly in low‐ and middle‐income countries (LMICs), where most people with dementia live.^1^

Previous research, predominantly conducted in high‐income countries, shows that women have a greater lifetime risk of developing dementia than men.^2^, ^3^, ^4^ This is partially due to longer survival into older age,^2^, ^3^ although this may not fully account for the sex difference in dementia risk.^5^ There is a growing recognition of sex as an effect modifier for several diseases as well as their risk factors (RFs),^6^ but the evidence remains sparse for dementia.^6^ However, many studies have examined RFs for dementia by adjusting for sex as a covariate rather than explicitly testing for sex differences, leaving the question of whether RF effects differ by sex unanswered.^5^, ^7^ The 2020 Lancet Commission Report estimated that up to 40% of dementia cases could be attributed to 12 modifiable RFs: less education, hypertension, obesity, diabetes, depression, hearing impairment, smoking, excessive alcohol consumption, physical inactivity, low social contact, traumatic brain injury, and air pollution.^8^ Other common RFs, such as unfavorable lipids profile^9^ and the presence of apolipoprotein E (*APOE*) ε4 allele,^5^ are also recognized for dementia.

While women appear to be affected disproportionately, the dementia burden is also distributed unevenly by ethnic groups^10^ and geographical regions.^1^, ^2^, ^7^ Previous studies were also limited by small samples and low generalizability (across countries or continents). The burden of dementia is increasing around the world, particularly in LMICs.^11^ High‐quality data from LMICs remains scarce, with one cross‐sectional study suggesting that dementia prevention potential is higher in LMICs, given that the RFs are more common in these regions.^12^ However, the sex difference in dementia risk in LMICs is less well known.^7^

The present study aimed to estimate the sex‐specific risk of all‐cause dementia and the sex‐specific association between major modifiable and non‐modifiable RFs and dementia, using individual participant data from the large‐scale, truly globally representative Cohort Studies of Memory in an International Consortium (COSMIC).^13^

## 2 METHODS

### 2.1 The international COSMIC consortium

Twenty‐one studies of the COSMIC collaboration^14^ contributed to the current analysis, comprising 45,628 participants. Details of each cohort are presented in Table S1 in supporting information.

The current analyses excluded those with dementia at study baseline (*n* = 2559). Table S2 in supporting information describes the dementia definitions used at baseline for each study. Participants without their sex recorded at the study baseline (*n* = 44), and those without any follow‐up (*n* = 13,175), were also excluded. A total of 28.9% of participants were lost to follow‐up; sex‐specific attrition rates for each study were calculated and are presented in Table S3 in supporting information, and characteristics by prespecified subgroups of those individuals lost to follow‐up are presented in Table S4 in supporting information. A total of 29,850 eligible participants were included in the study.

The 21 contributing cohorts varied in the number of participants (519 to 3237), location of the study (Africa: the Central African Republic, Republic of Congo, Nigeria; Asia: China, Japan, Malaysia, Singapore; Europe: France, Germany, Greece, Italy, Netherlands, Spain, Sweden; North America: United States; Oceania: Australia; South America: Brazil), all‐cause dementia outcome definition, the participation of women (48.5% to 75.4%), and year of baseline assessment (1993 to 2016).

### 2.2 Outcome definition and harmonization

All‐cause dementia, encompassing all subtypes of dementia, was used as the study outcome. This umbrella definition was used because of the variability in dementia definition across the studies, the small number of events by known dementia subtypes, and also considering that many dementia cases often have mixed neuropathology.^15^ All‐cause dementia was harmonized based on the dementia definition determined by the original cohorts and by the COSMIC harmonization protocol.^13^ This included either stand‐alone or a combination of: (1) batteries of neurocognitive tests, (2) clinical diagnosis based on Diagnostic and Statistical Manual of Mental Disorders (DSM) criteria (version III or IV), (3) clinical interviews (including the use of Clinical Dementia Rating scale) and the process is described in greater detail in Table S5 in supporting information.

In cases for which the exact times of the dementia diagnosis were unknown, given the variations in follow‐up intervals between cohorts, they were estimated by taking the mid‐point of the interval between the previous study visit and the visit when dementia was first recorded.^16^ Those without dementia recorded were censored at the end of study follow‐up or at the last study visit.

**RESEARCH IN CONTEXT**

1. **Systematic Review**: The authors reviewed the literature using traditional (e.g., PubMed) sources. Evidence around sex difference in dementia risk, and in the associations between risk factors (RFs) and dementia remains limited across diverse ethno‐regional groups.
2. **Interpretation**: Our findings suggest all‐cause dementia risk was higher in women than men, with possible variations by country‐level income settings and geographical regions, but there was no evidence of sex differences in most RFs, with the exceptions of longer education and former alcohol use and dementia, which showed stronger association with dementia risk for men than women.
3. F**uture Directions**: Our findings highlighted the importance for ongoing efforts to support programs to improve sex and gender equity in brain health, particularly in underrepresented populations. Future studies should investigate whether the modification of RFs might lessen the risk of dementia and disaggregate analyses by sex to clarify any differences, in diverse ethno‐regional populations.

### 2.3 Exposure variables

From the twelve RFs identified in the Lancet Commission 2020 report,^8^ we were able to harmonize nine RFs in the present analyses: baseline education years (Table S6 in supporting information); blood pressure indices (systolic and diastolic blood pressure, and hypertension [present if systolic blood pressure ≥140 mmHg and/or diastolic blood pressure ≥90 mmHg, received treatments for hypertension, self‐reported hypertension, or based on clinical diagnosis; Table S7 in supporting information]); obesity, using measured body anthropometry indicators (body mass index [BMI], waist circumference, and hip circumference); diabetes (present if fasting blood glucose criteria are ≥126 mg/dL or > 7 mmol/L, received treatments for diabetes, self‐reported diabetes, or based on clinical diagnosis; Table S8 in supporting information); depression (present if having current depressive symptoms [based on assessment tests cut‐offs], ever received current treatment for depression, history of depression or self‐reported depression [Table S9 in supporting information]); hearing impairment (present if self‐reported hearing loss, or based on clinical evaluation and interviewer's judgement [Table S10 in supporting information]); smoking status (defined as never, former, or current smoker); alcohol use (defined as never, former, or current alcohol use; Table S11 in supporting information); and physical activity (deemed either meeting World Health Organization recommended level or not for adults aged 65 years and above, detailed in Table S12 in supporting information). Additionally, baseline age (in years), *APOE* genotype was classified as: *APOE* ε3/ε3 (reference), *APOE* ε4 carriage (ε3/ε4 or ε4/ε4), or *APOE* ε2 carriage (ε2/ε2 or ε2/ε3); individuals with *APOE* ε2/ε4 genotype were excluded from the analyses, and cholesterol (total, high density lipoprotein [HDL], low density lipoprotein [LDL] cholesterol, triglycerides, and high cholesterol [present if total cholesterol ≥6.2 mmol/L and received lipid‐lowering treatments; Table S13 in supporting information]) were also analyzed.

### 2.4 Statistical analysis

Characteristics of women and men were summarized as number (percentage) for categorical variables and as mean (standard deviation) for continuous variables, unless they had skewed distributions when median (interquartile interval) was used.

Sex‐specific Kaplan–Meier survival curves were constructed using the survminer R package to assess the dementia‐free survival probability, and the log‐rank test was performed to compare the difference between the two survival curves. Only age and education were available for all individuals. Missing values (Table S14 in supporting information) were imputed using multiple imputation by chained equations (MICE), with 30 imputed datasets created using the mice R package in the pooled data from the 21 cohorts,^17^ on all exposures, covariates, and study outcomes. Different imputation methods were specified depending on the type of the variable (predictive mean matching [“pmm”] for numeric data, and logistic regression imputation [“logreg”] for binary data). The patterns of missingness were assessed using “md.pattern()” package in R.

Cox proportional hazards models were applied to calculate age‐ and education‐adjusted hazard ratios (HRs) with 95% confidence intervals (CIs) for sex as a RF (women vs. men) associated with the risk of all‐cause dementia in each study. A pooled estimate across all studies was calculated using a one‐stage meta‐analytical approach, combining the imputed individual participant data from 21 cohorts, using a Cox proportional hazards mixed effect model containing Gaussian random effects to take the study variability into account, using the R package coxme.^18^

Pooled sex‐specific, age‐ and education‐adjusted HRs were calculated for the associations between each RF and the risk of all‐cause dementia, using the one‐stage meta‐analysis approach using mixed‐effect Cox regression models accounting for the study as a random effect. Similarly, women‐to‐men ratio of hazard ratios (RHRs), were obtained by fitting the interaction term between each RF and sex.^19^ In a further sensitivity analysis, a complete case analysis was also applied.

We additionally estimated the sex‐specific, age‐adjusted incidence rates per 1000 person‐years, using Poisson regression models.

Subgroup analyses were conducted to explore whether sex differences in dementia risk and rate varied by prespecified subgroups: age at baseline (≥ or < 80 years; given the sex difference in dementia incidence has been reported to diverge after 80 years of age^5^, ^7^, ^20^), education years (≥ or < 9 years), birth cohorts (born before 1925, 1925 to 1934, 1935 and after; to yield roughly equal number of participants in each grouping), country‐level income (high‐income economies [gross national income (GNI) per capita of USD $12,696 or more]), upper middle‐income economies (between $4096 and $12,695), or low to lower middle‐income economies ($4095 or less) categorized by the 2022 fiscal year World Bank classification, region (Western countries, Asian countries, and other), and *APOE* genotype.

Additional analysis for *APOE* genotype examined the effect modifications on the sex differences by age and region.

Multiple‐adjusted pooled estimates were also produced from mixed‐effect Cox regression models, with different prespecified adjustment sets which excluded potential mediators, for each exposure.

Given the heterogeneity in incident dementia definitions, with the most common definition being DSM‐based across the cohorts (15 out of 21), sensitivity analysis was conducted by excluding the cohorts not using DSM criteria for defining dementia. The overall sex difference in dementia risk, and the sex differences in RF associations with dementia were evaluated.

All analyses were performed using R version 4.1.0.

### 2.5 Standard protocol approvals, registrations, and patient consent

This project was approved by the University of New South Wales Human Research Ethics Committee (HC 17292). Individual contributing cohorts obtained prior ethics approvals (Table S15 in supporting information).

## 3 RESULTS

### 3.1 Study characteristics

We included 29,850 eligible participants (58% women) without baseline dementia from 21 studies, representing 18 countries in six continents. A total of 2089 all‐cause dementia cases (66% women), over a median of 4.6 years of follow‐up (range: 0.01 to 19.6 years), were recorded. A total of 1442 dementia cases were recorded in 16,744 participants from Western countries (8.6%), 306 cases in 8031 participants from Asian countries (3.8%), and 341 cases recorded in 5075 participants from other countries (6.7%).

The mean age at baseline was 71.6 years (range: 24 to 120 years; and 72.0 years for women and 71.0 years for men; Table 1). On average, women had fewer years of education, and were more likely to have ever had depression; while men were more likely to have ever smoked, be currently consuming alcohol, and engaged in high physical activity, than women. Baseline characteristics for individual cohorts are presented in Table S16 in supporting information.

**TABLE 1:** Characteristics of combined participants by sex from 21 COSMIC cohorts

	Women (*N* = 17,295)	Men (*N* = 12,555)	Total (*N* = 29,850)
All‐cause dementia (*N*, %)	1372 (7.9)	717 (5.7)	2089 (7.0)
Age (years)	72.0 (9.5)	71.0 (9.9)	71.6 (9.6)
Education (years)	7.7 (5.0)	9.0 (5.2)	8.2 (5.1)
Blood pressure:			
Systolic blood pressure (mmHg)	139.8 (21.7)	140.9 (21.1)	140.3 (21.4)
Diastolic blood pressure (mmHg)	78.8 (11.9)	80.0 (11.7)	79.3 (11.8)
Hypertension (*N*, %)	10,287 (59.5)	7170 (57.1)	17,457 (58.5)
Body anthropometry:			
Body mass index (kg/m^2^)	26.4 (5.4)	25.9 (4.5)	26.2 (5.1)
Waist circumference (cm)	89.8 (13.2)	95.8 (12.7)	92.3 (13.3)
Hip circumference (cm)	101.1 (11.6)	100.0 (9.9)	100.7 (11.0)
Lipids:			
Total cholesterol (mmol/l)	5.4 (1.5)	5.0 (1.5)	5.2 (1.5)
HDL cholesterol (mmol/l)	1.5 (0.4)	1.3 (0.3)	1.4 (0.4)
LDL cholesterol (mmol/l)	3.4 (1.0)	3.2 (1.0)	3.3 (1.0)
Triglycerides (mmol/l) (Median (Q1, Q3)	1.9 (1.1, 3.2)	1.8 (1.1, 2.9)	1.8 (1.1, 3.1)
High cholesterol (*N*, %)	4571 (26.4)	2340 (18.6)	6911 (23.2)
Health conditions:			
Diabetes (*N*, %)	3107 (18.0)	2416 (19.2)	5523 (18.5)
Depression (*N*, %)	4351 (25.2)	1991 (15.9)	6342 (21.2)
Hearing impairment (*N*, %)	1215 (7.0)	833 (6.6)	2048 (6.9)
Lifestyle factors:			
Smoking:			
Current smoker (*N*, %)	695 (4.0)	1644 (13.1)	2339 (7.8)
Former smoker (*N*, %)	1202 (6.9)	3363 (26.8)	4565 (15.3)
Alcohol use:			
Current drinker (*N*, %)	5834 (33.7)	6925 (55.2)	12,759 (42.7)
Former drinker (*N*, %)	1230 (7.1)	932 (7.4)	2162 (7.2)
High physical activity (*N*, %)	6783 (39.2)	5850 (46.6)	12,633 (42.3)
*APOE* genotype:			
*APOE* ε2 carriage (*N*, %)	983 (5.7)	684 (5.4)	1667 (5.6)
*APOE* ε4 carriage (*N*, %)	1673 (9.7)	1072 (8.5)	2745 (9.2)

*Note*: Presented as mean (SD) unless stated otherwise.

Abbreviations: *APOE*, apolipoprotein E; HDL, high density lipoprotein; LDL, low density lipoprotein; SD, standard deviation.

### 3.2 Sex differences in the rates of all‐cause dementia

**FIGURE 1:**
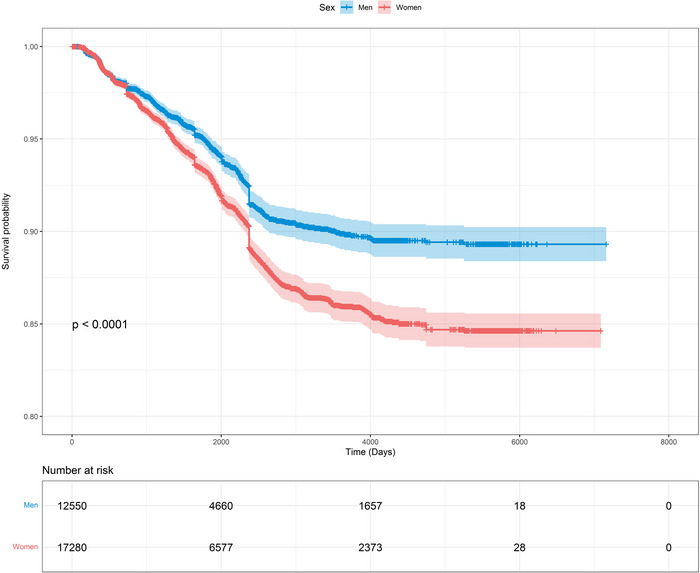
Sex‐specific Kaplan–Meier survival curve (and 95% confidence intervals) for survival probability of all‐cause dementia

Kaplan–Meier survival curves indicated that men survived longer without a diagnosis of dementia than women (log‐rank *P*‐value < 0.0001; Figure 1).

The age‐adjusted incidence rates (95% CIs) per 1000 person‐years for dementia were 16.4 (15.2, 17.6) in women and 12.3 (11.1, 13.5) in men (Table 2). Consistently, the incidence rates were higher in women than men across all the categories of the subgroups considered. Comparison between the subgroup categories revealed that higher incidence rates were observed among those ≥80 years than those <80 years, among those with *APOE* ε4 carriage than other *APOE* genotypes, and in cohorts of people born before 1925 than those born after. Interestingly, when stratified by country‐level income, incidence rates were the highest among women from low to LMICs, but rates were similar across country‐level incomes for men (Table 2).

**TABLE 2:** Age‐adjusted incidence rates of dementia (per 1000 person‐year)

	Rates/1000 person years (95% CI)	
	Women	Men
Overall	16.4 (15.2, 17.6)	12.3 (11.1, 13.5)
Baseline age:		
<80 years	9.8 (8.8, 10.7)	8.0 (6.9, 9.0)
≥80 years	43.0 (38.5, 47.6)	34.3 (29.3, 39.3)
Education years:		
≥9 years	16.7 (15.0, 18.5)	11.9 (10.2, 13.6)
<9 years	16.2 (14.5,17.8)	12.8 (11.0, 14.6)
Country‐level economies:		
High income	17.4 (15.9, 19.0)	13.7 (12.0, 15.4)
Upper‐middle income	13.1 (11.1, 15.1)	9.6 (7.6, 11.6)
Low to lower‐middle income	26.3 (19.8, 32.8)	13.8 (9.0, 18.5)
Region:		
Western countries	19.2 (17.5, 20.9)	14.8 (13.0, 16.7)
Asian countries	14.4 (11.6, 17.1)	10.2 (7.6, 12.8)
Other	13.1 (10.8, 15.3)	9.0 (6.9, 11.2)
*APOE* genotype:		
ε3/ε3	12.2 (10.4, 13.9)	10.9 (8.8, 12.9)
ε2 carriage	13.8 (9.6, 17.9)	10.7 (6.3, 15.2)
ε4 carriage	24.6 (20.3, 28.9)	16.3 (12.2, 20.5)
Birth cohort:		
After 1934	6.9 (5.6, 8.3)	6.5 (5.0, 7.9)
1925 to 1934	14.2 (12.3, 16.1)	12.5 (10.4, 14.6)
Before 1925	33.9 (30.6, 37.2)	25.1 (21.4, 28.7)

Abbreviations: *APOE*, apolipoprotein E; CI, confidence interval.

### 3.3 Sex differences in the risk of all‐cause dementia

**FIGURE 2 alz12962-fig-0002:**
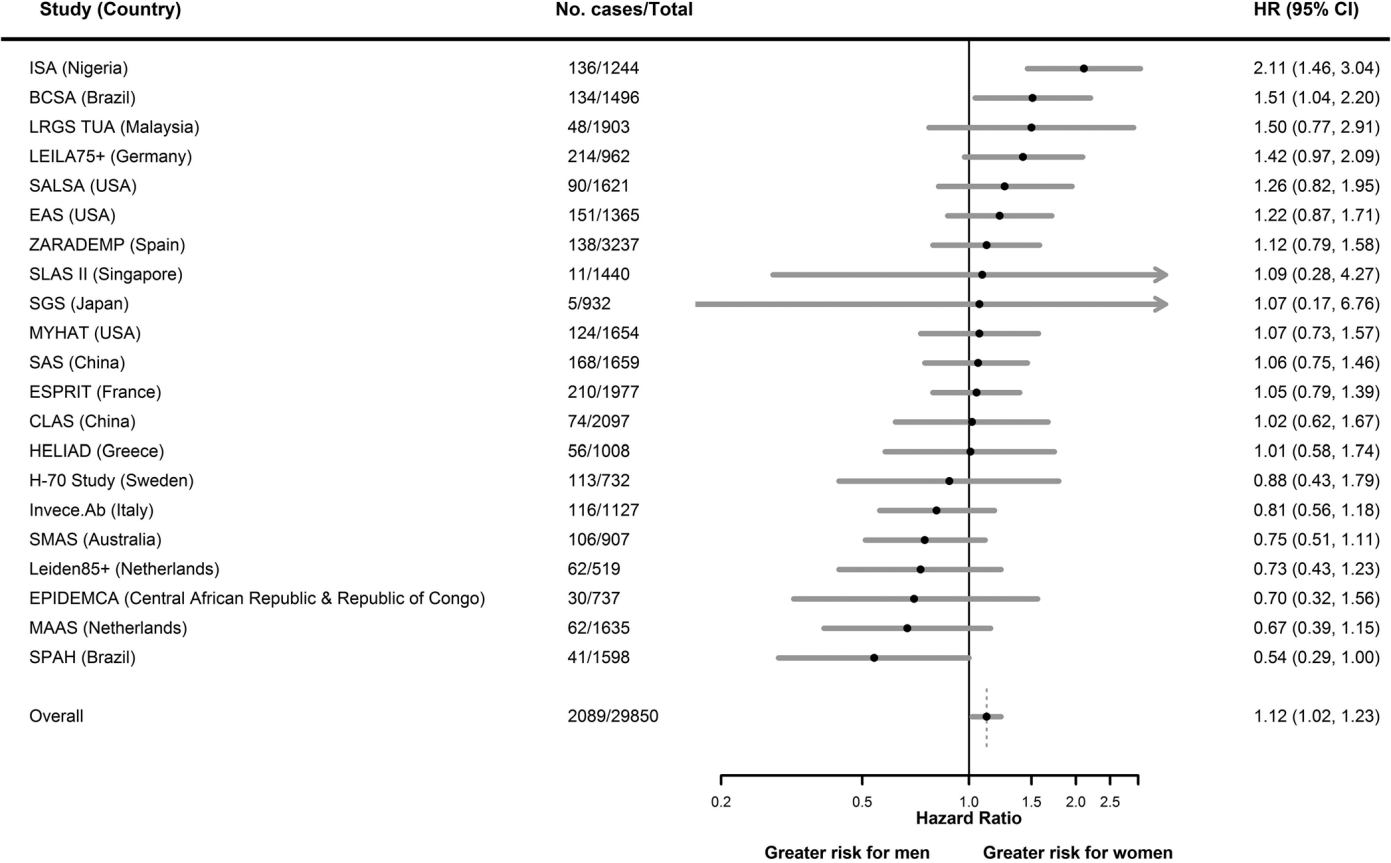
Age‐ and education‐adjusted study‐specific and pooled hazard ratios for all‐cause dementia by sex (women vs. men). CI, confidence interval; HR, hazard ratio

The pooled risk of all‐cause dementia was higher in women than men after adjusting for age and education (HR, 1.12 [1.02, 1.23]), although not uniform across the individual cohorts (Figure 2), of which 14 cohorts reported the risk to be higher in women (from Nigeria, Brazil, Malaysia, Germany, United States, Spain, Singapore, Japan, China, France), and 7 cohorts reported the opposite (from Greece, Sweden, Italy, Australia, Netherlands, Central African Republic, Republic of Congo, Brazil), although for many the 95% CIs crossed unity.

**FIGURE 3 alz12962-fig-0003:**
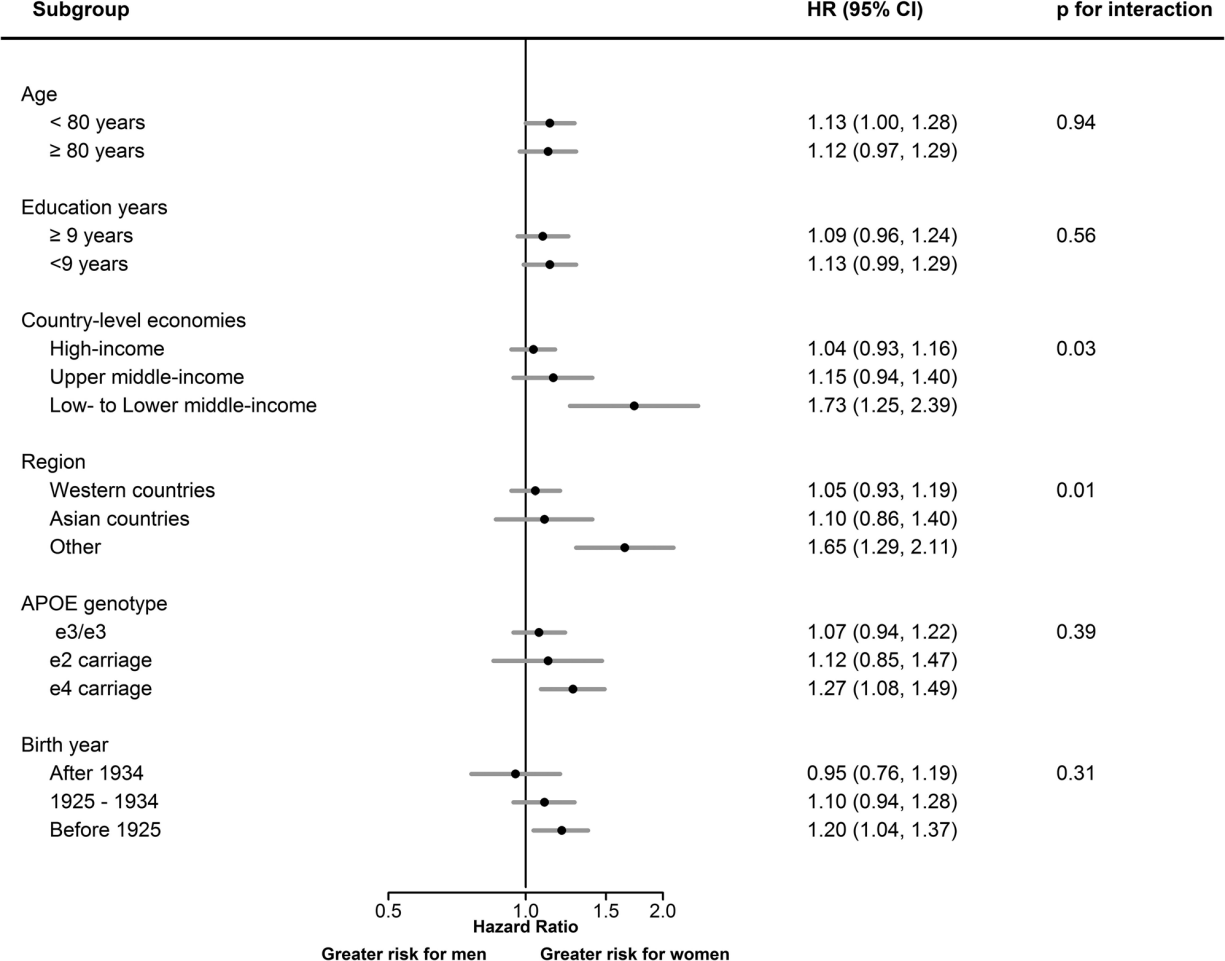
Age‐ and education‐adjusted pooled hazard ratios for sex (women vs. men) in association with all‐cause dementia, stratified by subgroups. *APOE*, apolipoprotein E; CI, confidence interval; HR, hazard ratio

When examined by subgroup (by baseline age (≥ or < 80 years), education years (≥ or < 9 years), birth cohorts, country‐level income, region, and *APOE* genotypes, the greater risk in women was most pronounced in low‐ to lower‐middle‐income economies (HR, 1.73 [1.25, 2.39]; *P* for interaction = 0.03). When examined by regions, the greater risk in women was observed in those from other (South America and Africa) countries (HR, 1.65 [1.29, 2.11]; *P* for interaction = 0.01), but neither in Western (HR, 1.05 [0.93, 1.19]) nor Asian countries (HR, 1.10 [0.86, 1.40]; Figure 3).

### 3.4 Sex differences in risk factor associations

**FIGURE 4 alz12962-fig-0004:**
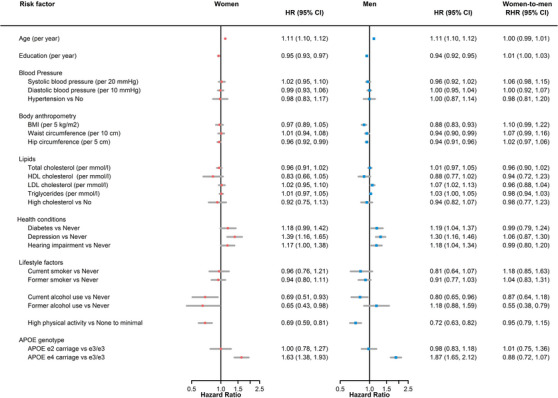
Age‐ and education‐adjusted women‐to‐men ratio of hazard ratios for all‐cause dementia by risk factor. *APOE*, apolipoprotein E; BMI, body mass index; CI, confidence interval; HDL, high density lipoprotein; HR, hazard ratio; LDL, low density lipoprotein; RHR, ratio of hazard ratios

In women and men, older age, diabetes, depression, hearing impairment, and *APOE* ε4 carriage were associated with a greater risk of dementia (Figure 4); while more years of education, higher hip circumference, current alcohol use (vs. never), and high physical activity (vs. none to minimal) were associated with a lower risk of dementia.

Lower BMI was associated with lower risk of dementia (HR, 0.88 [0.83, 0.93]), and higher LDL cholesterol was associated with higher risk of dementia (HR, 1.07 [1.02, 1.13]), in men, but these associations were not significant in women.

Among all RFs considered, sex differences were only clear for former alcohol use in association with dementia, with the relationship being stronger in men than women (women‐to‐men RHRs, 0.55 [0.38, 0.79]). Additionally, years spent in education showed moderate evidence for a sex difference (women‐to‐men RHRs, 1.01 [1.00, 1.03]), indicating a stronger protective effect for men than women.

There was no evidence of an association between *APOE* ε2 carriage (vs. ε3/ε3) and dementia risk in either sex (Figure 4). Further, there was little evidence indicating a sex difference (RHR, 0.88 [0.72, 1.07]), or an effect modification by age or region on the sex difference, in the effect of *APOE* ε4 carriage and all‐cause dementia (Table S17 in supporting information).

### 3.5 Sensitivity analyses

Estimates from a complete case analysis were similar to those using imputed data (Table S18 in supporting information). Furthermore, multiple‐adjusted estimates for both sex as a RF (HR, 1.10 [1.00, 1.20]), and sex‐specific estimates in RFs in association with dementia showed broadly similar findings, compared to the age‐ and education‐adjusted estimates (Table S19 in supporting information).

As a part of the sensitivity analysis, only the cohorts using a DSM‐based dementia definition were included (a total of 15 out of 21 cohorts). The sex difference in dementia risk was no longer significant (HR, 1.02 [0.91, 1.15]) after adjusting for age and education. The results for RF associations with dementia were broadly similar for both sexes (Table S20 in supporting information).

### Missing data

The variables included in the “md.pattern” function were age, education, systolic blood pressure, diastolic blood pressure, BMI, waist circumference, hip circumference, total cholesterol, HDL cholesterol, LDL cholesterol, triglycerides, diabetes, depression, hearing impairment, current smoking, current alcohol, and *APOE* ε4. This approach allowed us to look at the number missing for each variable, the type of missing data pattern, and the frequency of participants with each missing data pattern. A total of 779 participants (2.6%) did not have any variables missing. The missing data pattern combinations that occurred with >5% of participants were *APOE* ε4 and hearing impairment (5% of participants); hearing impairment, hip circumference, and waist circumference (5.3% participants); *APOE* ε4, hearing impairment, hip circumference, and waist circumference (6.8% participants); and *APOE* ε4, hearing impairment, hip circumference, waist circumference, physical activity, total cholesterol, HDL cholesterol, LDL cholesterol, and triglycerides (9.7% participants). More than 400 missing data patterns were observed; Table S21 in supporting information presents the missing data patterns for the 25 patterns that occur for > 1% of participants.[Fig alz12962-fig-0002], [Fig alz12962-fig-0003], [Fig alz12962-fig-0004]

## 4 DISCUSSION

In this individual participant data study from the international COSMIC consortium, the risk of all‐cause dementia was higher in women than men, although not uniform across cohorts. The age‐adjusted incidence rate per 1000 person‐years for dementia was higher in women than men, and the greater risk in women was more pronounced in LMICs, and in Africa or South America. There was some evidence indicating that the associations for more years spent in education and former alcohol use with dementia risk appear to be stronger for men than women.

### 4.1 Heterogeneity in sex‐specific risk of dementia

While the pooled estimate showed a greater risk of dementia in women than men, there were significant country‐level variations for the sex differences, with many cohorts not showing a sex difference in dementia risk, which may be due to insufficient statistical power. Several cohorts from Europe,^21^, ^22^ North America,^4^, ^21^, ^23^, ^24^, ^25^, ^26^, ^27^, ^28^, ^29^ and Latin America^30^ found a similar age‐specific incidence of dementia for women and men. Other studies in Europe^20^, ^31^, ^32^, ^33^, ^34^, ^35^ and in Asia^36^, ^37^ observed a higher incidence in women than men, with these differences most pronounced in those of 80 years of age and over.^5^, ^7^, ^20^ Our study found the age‐adjusted rate of dementia to be the highest among low‐ to lower‐middle‐income countries, and higher in women than men. Comparably, the 10/66 Study, with participants recruited from a number of LMICs (China, Cuba, Dominican Republic, Mexico, Peru, and Venezuela), also reported a higher age‐adjusted incidence in women.^38^ Women, particularly from LMICs, have not had equal educational and occupational opportunities,^5^, ^7^ and higher educational attainment and mentally stimulating occupations have been shown to be protective against dementia.^39^, ^40^ Structural discrimination, restricted access to appropriate health care and risk management programs,^7^ and other unmeasured factors such as domestic violence,^7^ particularly for women from lower socioeconomic settings, can further lead to downstream consequences such as increased psychological stress^3^ and worse financial positions,^7^ in turn affecting late‐life cognitive health. Nevertheless, the two Brazilian cohorts showed opposite findings, the Bambui (Brazil) Cohort Study of Ageing showed a greater risk of dementia in women, and the São Paulo Aging & Health Study cohort showed the opposite, indicating that there may be other underlying explanations for the sex difference observed in dementia risk across different populations, including within‐country variation (e.g., urban vs. rural residence, area deprivation, or race). Notably, one recent study using nationally representative data in Brazil concluded that the overall weighted population attributable fraction (PAF) of RFs for dementia was larger in poor regions than rich regions, but the overall weighted PAFs were similar between different races.^41^

After examining by birth year, which incorporated information on age and time periods, we found the sex difference in dementia risk was largely restricted to those born before 1925. While this could be a chance finding, the importance of examining dementia incidence by birth year has been previously highlighted,^42^, ^43^, ^44^ and the influence of early‐life environment and societal factors embedded in historical context need to be taken into consideration.^45^ Women and men may experience parenthood^46^ and financial hardship^5^, ^7^ differently, possibly mediated by the aforementioned differences in educational and occupational factors.^5^, ^7^ These different experiences may be more extreme in those born in the early 20th century, given the adverse global events which took place during that time.^42^, ^44^ Nevertheless, population brain health may be shifting around the world,^47^ akin to progress in achieving sex and gender equity,^48^ and increased effort in equitable cardiovascular disease prevention^21^, ^47^ and improved social welfare on a wider population level.

### 4.2 Sex differences in risk factor associations

The current study found similar associations between most RFs and dementia risk in men and women, and there was only moderate evidence indicating that *APOE* ε4 carriage was more strongly associated with dementia risk in men than women in our diverse populations. In contrast, previous studies from the United States reported a stronger association between *APOE* ε4 and dementia risk for women than men.^49^, ^50^ In our global dataset, we found no evidence indicating an effect modification by age or region, on the sex differences in the effect of *APOE* ε4 carriage on all‐cause dementia. A previous meta‐analysis of 27 independent studies, with data representative of non‐Hispanic White individuals in North America and Europe, found that the effect of a single copy of *APOE* ε4 on Alzheimer's disease (AD) was similar for women and men. The study also found some evidence for effect modification by age in those with one copy of *APOE* ε4, such that women aged 65 to 75 years were at an increased risk of AD than men of the same age group.^51^ Nevertheless, particular caution is warranted when interpreting sex‐specific genetic associations, given the differential sex survival distributions and that the *APOE* gene may have pleiotropic effects (potentially influencing both the risk of dementia and mortality/longevity), as well as the high level of missingness for *APOE* genotypes, which can introduce spurious associations, even if there are no true sex differences present.^52^

Another COSMIC study examined the relationship between alcohol and dementia,^53^ and found that abstinence from alcohol was associated with a greater risk of all‐cause dementia, similarly for women and men. Our finding that former, but not current, alcohol use is more strongly related to dementia in men than women is intriguing and requires further study, but could be due to self‐deception in self‐reported alcohol use.

Notwithstanding the absence of a sex difference in most of the RFs associated with dementia studied here, which is largely inconsistent with a previous study in the Nordic populations,^5^ sex differences in the prevalence of the RFs may also drive the sex differences seen in dementia risk. A COSMIC study found that early life education and adulthood occupational complexity were linked to dementia independently.^40^ However, the study did not find any sex difference, which may be, at least in part, due to the exclusion of participants without lifetime occupation.^40^ As previously discussed, low education and social disadvantage remain more pervasive among women than men around the world.^7^ There was moderate evidence indicating more years spent in education were more protective for men than women in terms of dementia risk, with previous studies also showing corroborating findings,^54^, ^55^ and the extent to which this could be attributed to sex difference in cognitive reserve needs further investigations.^5^ Given that education has been consistently linked to dementia for both women and men, this represents a significant missed opportunity in dementia prevention, given the unequal access to education in many parts of the world, particularly in girls.^12^ Importantly, how early‐life socioeconomic disadvantage is manifested in brain health later in life,^10^ and how it interacts with cardiovascular RFs, and may be related to sex differences in dementia risk need to be better contextualized.

Sex‐specific RFs, including reproductive factors and the hormonal milieu, may also be pertinent in understanding the differential contributions to the sex‐specific risk of dementia.^56^ These should be considered when interpreting the findings from the current study, given that the women included are likely to be post‐menopausal. A recent study using UK Biobank data also found several reproductive events related to shorter cumulative exposure to endogenous estrogen were linked to a greater risk of dementia.^46^ Future studies with repeated measures of sex hormones, together with reliable documentation of sex‐specific factors and exogenous hormone use through the life course would be necessary to understand their contributions to the sex‐specific risk of dementia.

### 4.3 Strengths and limitations

This study was strengthened by the large, ethno‐regionally diverse underlying populations, with data from 18 countries across six continents, with many from traditionally underrepresented research populations and study settings, enhancing the potential generalizability in a broader geographical context. Another major strength was the harmonization of RFs across the cohorts, which allowed us to systematically examine sex differences for a comprehensive list of RFs.

Limitations of the study should be acknowledged. Given the variations in assessment intervals across the studies, the exact time of diagnosis of dementia may not be reliably estimated in each study. While we excluded all dementia cases, as defined by the original study, at study baseline, mild cognitive impairment was not excluded as it is not uniformly recorded for all cohorts included. Hence, some prodromal dementia cases may be missed. Another limitation is the lack of examination by dementia subtypes, and because sex differences have been reported in previous literature by AD and vascular dementia, the combination of all‐cause dementia may mask the emergence of some of these differences. Reverse causation in some of the risk exposures is inherent in many observational studies, and it can be challenging when the study outcome is dementia, given its insidious onset, and hence our findings do not imply causality; nevertheless, the exclusion of baseline dementia allowed us to minimize the effect of this bias. Other unmeasured factors such as air pollution, social interactions, frailty, and the use of post‐menopausal hormone therapy mean residual confounding could be possible, and raise the possibility of ascertainment bias, particularly in LMICs. Further, despite our best effort, certain factors are difficult to harmonize; for example, the measure of years spent in education may mean different things depending on whether compulsory schooling is in place in a country. There was also heterogeneity in dementia definition across the cohorts. A major limitation is that several COSMIC cohorts relied on using cognitive screening tests to define dementia, rather than establishing a clinical diagnosis. This is likely to introduce potential misclassification of dementia cases. In further sensitivity analyses with cohorts using DSM criteria for defining dementia cases, the greater risk of dementia in women compared to men was no longer significant. Another notable limitation was that most of the exposures were measured in mid‐life and late life, though factors involved in early life may also contribute to differential risk for women and men. Future studies should consider taking a life‐course approach to study the RFs for dementia.^57^ Last, we could not account for the competing risk of mortality in our analysis.

## 5 CONCLUSION

In summary, in this study of diverse global populations, a greater risk of dementia for women was observed, contributing to the accruing evidence for sex differences in dementia risk. Although there was almost no evidence of sex differences in most RFs for dementia, the excess risk in women was more pronounced in poorer countries, suggesting possible geographical variations following sex disparity in general terms. Given the diverse socio‐political, socioeconomic, and cultural contexts across the world, these findings justify ongoing efforts to support programs to improve sex and gender equity in brain health throughout the life course, particularly in the populace from underrepresented settings.

## AUTHOR CONTRIBUTIONS

Conception and design of the study: Jessica Gong and Mark Woodward conceived the current study. Jessica Gong, Mark Woodward, Katie Harris, Sanne A. E. Peters, Maree L. Hackett, and Henry Brodaty were involved in the study design. Acquisition of data: COSMIC consortium committee. Analysis of data and statistical analysis: Jessica Gong had full access to all the data included in the study and hence responsibility for all statistical analyses. Jessica Gong carried out all statistical analyses, with support and supervision from Mark Woodward, Katie Harris, Darren M. Lipnicki, Sanne A. E. Peters, and Maree L. Hackett. Drafting of the manuscript, figure, and tables: Jessica Gong wrote the first draft of the manuscript, and generated all figures and tables included in the manuscript. Critical revision of the manuscript: All authors critically revised the manuscript and were involved in data interpretation.

## CONFLICTS OF INTEREST

B.S.D. has received payment or honoraria for lectures, presentations, speakers bureaus, manuscript writing, or educational events from UOL Tecnologia Educacional, Brazil; and participates on a Data Safety Monitoring Board or Advisory Board for Depression treatment and Aβ dynamics: A study of Alzheimer's disease risk (ABD Study) (1 R01 AG070821‐01A1). R.B.L. received funding support from NIH/NIA 2PO1 AG003949 (Einstein Aging Study), S&L Marx Foundation, Czap Foundation; grants from the FDA, the Migraine Research Foundation, and the National Headache Foundation; served as consultant, advisory board member, and received honoraria from or research support from: Abbvie (Allergan), American Academy of Neurology, American Headache Society, Amgen, Biohaven, Biovision, Boston, Dr. Reddy's (Promius), Electrocore, Eli Lilly, eNeura, Equinox, GlaxoSmithKline, Grifols, Lundbeck (Alder), Merck, Pernix, Pfizer, Teva, Vector, and Vedanta; and has stock in Biohaven and Manistee. M.J.K. is supported by NIH/NIA AG03949. C.W. is supported by NIH/NIA AG003949. M.G. is supported by French National Research Agency, AXA Research Fund, and Limoges University Hospital (France). N.S. is supported by Alzheimer's Association grant IIRG‐09‐133014, European Social Fund grants 189 10276/8/9/2011, National Strategic Reference Framework‐EU program Excellence Grant (ARISTEIA), and Greek Ministry of Health grants DY2b/oik.51657/14.4.2009, as well as EPAD—Local PI of recruiting site for multinational, multicenter Innovative Medicines Initiative (IMI) sponsored observational study of prodromal stages of dementia, and NovoNordisc—Local PI of recruiting site for multinational, multicenter industry sponsored phase III treatment trial for Alzheimer's disease; and served on the Chair of Data Safety Monitoring Board for Albert Einstein College of Medicine—NIH funded study. M.Y. is supported by European Social Fund and Ministry of Health, and received fundings from ERASMUS+—European Commission and HORIZON2020 – European Commission; served as the President of the National Nutrition Policy Committee – no fees. M.G. is supported by the National Institute on Aging, NIH, received payments from the University of Connecticut Health Center for honoraria for lectures; participated on a Data Safety Monitoring Board or Advisory Board for Indiana University School of Medicine; and received honorarium payment from the *Journal of the American Geriatrics Society*. C.C.C. is supported by the NIH (R01 grant: MYHAT study). A.L. and ZARADEMP study was supported by grants from the Fondo de Investigación Sanitaria, Instituto de Salud Carlos III, Spanish Ministry of Economy and Competitiveness, Madrid, Spain (grants 94/1562, 97/1321E, 98/0103, 01/0255, 03/0815, 06/0617, G03/128, 12/02254, 16/00896, 19/01874), and the Fondo Europeo de Desarrollo Regional (FEDER) of the European Union and Gobierno de Aragón, (grant B15_17R). Group #19; A.L. received financial support to attend scientific meeting from Janssen. E.L. has received honorarium from University of Granada. None of these activities was related to the current project. M.W. is supported by Australian NHMRC and EU Horizon 2020, and is a consultant for Amgen and Freeline outside the submitted work; and participated on a Data Safety Monitoring Board or Advisory Board for DMB STAREE trial (no payment). The rest of the authors have no conflicts of interest. C.D.L.C. has received financial support to attend scientific meetings from Janssen, Almirall, Lilly, Lundbeck, Rovi, Esteve, Novartis, Astrazeneca, Pfizer, and Casen Recordati. M.W. has been a consultant for Amgen, Freeline, and Kirin outside the submitted work. Author disclosures are available in the supporting information.

## Supporting information

Supplementary Information

Supplementary Information
