# Supplementary material for: Sex differences in dementia risk and risk factors: Individual‐participant data analysis using 21 cohorts across six continents from the COSMIC consortium
Source: Alzheimers Dement. 2023 Feb 15;19(8):3365–78. doi: 10.1002/alz.12962 (PMC10955774; doi:10.1002/alz.12962)
Supplement: Supplementary file 1 — Supplementary Information [file ALZ-19-3365-s001.docx]

**Supplementary Table 1: Study characteristics of included COSMIC studies.**

| **Study** | **Area, Country** | **Region** | **World Bank income classification ^1^** | **No. of participants included** | **Assessment waves** | **Study period** | **Mean age, years (SD)** | **Women, N (%)** | **Incident dementia, N (%)** |
| --- | --- | --- | --- | --- | --- | --- | --- | --- | --- |
| Bambui Cohort Study of Ageing (BCSA) | Bambui, Brazil | South America | Upper-middle-income economies | 1496 | Baseline; annual follow-up | 1997 – 2013 | 69.0 (7.1) | 912 (61.0) | 134 (9.0) |
| Chinese Longitudinal Aging Study (CLAS) | Beijing, Shanghai, Zhejiang, Shanxi, Liaoning, Jiangxi, Guangdong, and Anhui, China | Asia | Upper-middle-income economies | 2097 | Baseline; 1-, 3-, and 5-years follow-up | 2011 – | 71.4 (7.9) | 1133 (54.0) | 74 (3.5) |
| Einstein Aging Study (EAS) | New York, USA | Western countries | High-income economies | 1365 | Baseline; annual follow-up | 1993 – 2017 | 78.0 (5.4) | 840 (61.5) | 151 (11.1) |
| Epidemiology of Dementia in Central Africa (EPIDEMCA) | Central African Republic & Republic of Congo | Africa | Low-income economies | 737 | Baseline; 2 years follow-up | 2011 – 2014 | 73.6 (6.2) | 429 (58.2) | 30 (4.1) |
| Etude Santé Psychologique Prévalence Risques et Traitement (ESPRIT) | Montpellier, France | Western countries | High-income economies | 1977 | Baseline; 2-, 4-, 7- and 10-years follow-up | 1999 – | 72.9 (5.4) | 1162 (58.8) | 210 (10.6) |
| The Gothenburg H70 Birth Cohort Study (H70-study) | Gothenburg, Sweden | Western countries | High-income economies | 732 | Baseline; Follow-up at ages 75, 79, 81, 83, 85, 88, 90, 92, 95, 97, 99 and 100 years | 2000 – 02, 2005 – 06, 2009 – 11 | 73.6 (5.0) | 552 (75.4) | 113 (15.5) |
| Hellenic Longitudinal Investigation of Aging and Diet (HELIAD) | Larisa/Marousi, Greece | Western countries | High-income economies | 1008 | Baseline; every 3-year follow-up | 2010 – | 72.6 (5.3) | 602 (59.7) | 56 (5.6) |
| Invecchiamento Cerebrale in Abbiategrasso (Invece.Ab) | Abbiategrasso, Italy | Western countries | High-income economies | 1127 | Baseline; every 2-year follow-up | 2010 – 2015 | 72.2 (1.3) | 608 (53.9) | 116 (10.3) |
| Ibadan Study of Aging (ISA) | Ibadan, Nigeria | Africa | Low-middle income economies | 1244 | Baseline; follow-up in 2007 | 2003/04 – 2007 | 74.1 (8.3) | 639 (51.4) | 136 (10.9) |
| Leiden 85-plus Study (Leiden85+) | Leiden, the Netherlands | Western countries | High-income economies | 519 | Baseline; every 1-year for 5 years | 1997 – 1999 | 85 (0.0) | 350 (67.4) | 62 (11.9) |
| Leipzig Longitudinal Study of the Aged (LEILA75+) | Leipzig, Germany | Western countries | High-income economies | 962 | Baseline; 5 follow-ups with 1.5-year intervals, and 6^th^ follow-up after 8 years. | 1997 – 2014 | 81.5 (4.9) | 712 (74.0) | 214 (22.2) |
| Neuroprotective Model for Healthy Longevity among Malaysian Older Adults (LRGS TUA) | Johor, Perak, Selangor and Kelantan, Malaysia | Asia | Upper-middle-income economies | 1903 | Baseline, 18 and 36 months, 5 years | 2012/3 - | 68.7 (6.0) | 972 (51.1) | 48 (2.5) |
| Maastricht Aging Study (MAAS) | South Limburg, the Netherlands | Western countries | High-income economies | 1635 | Baseline; Follow-up after 6 and 12 years | 1993 - 2010 | 51.6 (16.2) | 793 (48.5) | 62 (3.8) |
| Monongahela-Youghiogheny Healthy Aging Team (MYHAT) | Pennsylvania, USA | Western countries | High-income economies | 1654 | Baseline; annual follow-ups (the 8th in 2014-16) | 2006/08 – | 77.4 (7.3) | 1029 (62.2) | 124 (7.5) |
| Sacramento Area Latino Study on Aging (SALSA) | Sacramento area, CA, USA | Western countries | High-income economies | 1621 | Baseline; Every 12 to 15 months, for a maximum of 6 follow-ups | 1998 – 2008 | 70.2 (6.7) | 941 (58.1) | 90 (5.5) |
| Shanghai Aging Study (SAS) | Shanghai, China | Asia | Upper-middle-income economies | 1659 | Baseline; Follow-up: 4 years | 2010 – | 71.5 (7.4) | 900 (54.2) | 168 (10.1) |
| Sasaguri Genkimon Study (SGS) | Sasaguri, Japan | Asia | High-income economies | 932 | Baseline; Follow-up: 2 years | 2011 – 2013 | 72.7 (5.6) | 521 (55.9) | 5 (0.54) |
| Singapore Longitudinal Ageing Studies (SLAS II) | Singapore | Asia | High-income economies | 1440 | Baseline, follow-ups 1-2 and 4 years | 2003 – | 65.5 (6.9) | 934 (64.9) | 11 (0.76) |
| Sydney Memory and ageing study (SMAS) | Sydney, Australia | Western countries | High-income economies | 907 | Baseline; every 2-year follow-up | 2005 – | 78.6 (4.8) | 493 (54.4) | 106 (11.7) |
| Sao Paulo Ageing & Health Study (SPAH) | São Paulo, Brazil | South America | Upper-middle-income economies | 1598 | Baseline; every 2-year follow-up | 2003 – 2008 | 71.6 (5.7) | 984 (61.6) | 41 (2.6) |
| ZARAgoza DEMentia DEPression Project (ZARADEMP) | Zaragoza, Spain | Western countries | High-income economies | 3237 | Baseline; 3, 5-year follow-up | 1994 – | 72.0 (8.8) | 1789 (55.3) | 138 (4.3) |

^1^ Based on 2022 fiscal year classification.

**Supplementary Table 2: Definition of all-cause dementia at baseline in each cohort.**

| **Study** | **Dementia definition** |
| --- | --- |
| BCSA | MMSE score cut-off point 13 appropriate for Brazilian populations with low schooling (deemed appropriate for Brazilian populations with low schooling)^1^ |
| CLAS | DSM-IV |
| EAS | DSM-IV |
| EPIDEMCA | Cognitive disorders were assessed at baseline and follow-up assessments through a two-phase screening and diagnostic procedure. The Community Screening Interview for Dementia (CSI-D) was administered to identify suspected cases of dementia. All participants obtaining a poor performance on the CSI-D (COGSCORE ≤ 24.5) were referred for detailed clinical assessment with a neurologist. A neurological examination was performed. Orientation skills and daily living activities were also investigated to evaluate dependence. Neurological assessments also involved additional cognitive tests. Consensus diagnosis was determined according to DSM-IV criteria and based on reviewing of all medical records and performance on clinical assessments and cognitive tests. |
| ESPRIT | Standardized interview by a neurologist incorporating cognitive testing, with diagnoses validated by an independent panel of expert neurologists |
| H70-study | Dementia was diagnosed following the Diagnostic and Statistical Manual of Mental Disorders DSM III-R criteria as closely as possible using information from the clinical cognition examination and information from key informants. |
| HELIAD | DSM-IV |
| Invece.Ab | DSM-IV |
| ISA | We excluded from the analysis anybody with probable dementia that had been diagnosed by a previously validated cognitive screen.^2^ The WMH-CIDI is a user-friendly version of the CIDI, with simplified questions and structure. Diagnosis was made with the same algorithm as in our earlier study,^3^ in which we used WMH-CIDI. |
| Leiden85+ | Severe cognitive impairment was defined as an MMSE score of 18 points or lower. |
| LEILA75+ | DSM-IV |
| LRGS TUA | MMSE ≤ 2 SD and IADL < 6 or Clinical Dementia Rating ≥ 1, if available |
| MAAS | At baseline, participants were excluded based on an existing diagnosis of dementia or a score below 24 on the Mini-Mental State Examination (MMSE). Included participants were assessed on a battery of cognitive tests, as well as activities of daily living and subjective cognitive complaints. A general practitioner and the investigators identified cases of dementia in accordance with DSM-III-R/DSM-IV criteria using all available clinical information and neuropsychological test data at all follow up occasions |
| MYHAT | Clinical Dementia Rating ≥ 1 |
| SALSA | DSM-IV |
| SAS | DSM-IV |
| SGS | Self-reported medical history |
| SLAS II | The absence of dementia was defined by the presence of (i) a modified Mini-Mental State Examination (MMSE) score more than 26, or (ii) MOCA score more than 26, or (iii) Clinical Dementia Rating (CDR) scale global score of 0, or (iv) CDR global score of 0.5 and Sum of Boxes score less than 3. |
| SMAS | DSM-IV |
| SPAH | DSM-IV |
| ZARADEMP | DSM-IV |

CDR, Clinical Dementia Rating; CIDI, Composite International Diagnostic Interview; COGSCORE, General Practitioner Assessment of Cognition Score; CSI-D, Community Screening Instrument for Dementia; DSM, Diagnostic and Statistical Manual of Mental Disorders; IADL, Instrumental Activities of Daily Living; MMSE, Mini–Mental State Examination; MoCA, Montreal Cognitive Assessment; WMH-CIDI; World Mental Health Composite International Diagnostic Interview.

1 Castro-Costa E, Fuzikawa C, Uchoa E, Firmo JO, Lima-Costa MF. Norms for the mini-mental state examination: adjustment of the cut-off point in population-based studies (evidences from the Bambui health aging study). Arq Neuropsiquiatr 2008;66:524-8

2 Gureje O, Ogunniyi A, Kola L. The profile and impact of probable dementia in a sub-Saharan African community: results from theIbadan Study of Aging. J Psychosom Res 2006; 61: 327–33.

3 Gureje O, Lasebikan VO, Kola L, Makanjuola VA. Lifetime and 12-month prevalence of mental disorders in the Nigerian Survey of Mental Health and Well-being. Br J Psychiatry 2006; 188: 465–71.

**Supplementary Table 3: Study-specific and overall attrition rates, by sex.**

| **Study** | **Total women at baseline** | **Women lost to follow up** | **Women lost to follow up/Women at baseline** | **Total men at baseline** | **Men lost to follow-up** | **Men lost to follow up/Men at baseline** | **Total participants at baseline** | **Overall lost to follow up** |
| --- | --- | --- | --- | --- | --- | --- | --- | --- |
|  | **N** | **N** | **%** | **N** | **N** | **%** | **N** | **N (%)** |
| BCSA | 913 | 1 | 0.1% | 584 | 0 | 0.0% | 1497 | 1 (0.1) |
| CLAS | 1642 | 509 | 31.0% | 1386 | 422 | 30.4% | 3028 | 931 (28.5) |
| EAS | 1335 | 494 | 37.0% | 824 | 300 | 36.4% | 2159 | 794 (34.7) |
| EPIDEMCA | 1131 | 688 | 60.8% | 736 | 442 | 60.1% | 1867 | 1130 (56.4) |
| ESPRIT | 1162 | 0 | 0.0% | 815 | 0 | 0.0% | 1977 | 0 (0.0) |
| H70-study | 697 | 0 | 0.0% | 224 | 189 | 84.4% | 921 | 189 (15.5) |
| HELIAD | 1176 | 574 | 48.8% | 782 | 376 | 48.1% | 1958 | 950 (46.1) |
| Invece.Ab | 684 | 76 | 11.1% | 583 | 64 | 11.0% | 1267 | 140 (10.6) |
| ISA | 969 | 330 | 34.1% | 925 | 320 | 34.6% | 1894 | 650 (30.2) |
| Leiden85+ | 397 | 47 | 11.8% | 202 | 33 | 16.3% | 599 | 80 (13.3) |
| LEILA75+ | 782 | 70 | 9.0% | 263 | 13 | 4.9% | 1045 | 83 (6.6) |
| LRGS TUA | 1208 | 236 | 19.5% | 1114 | 183 | 16.4% | 2322 | 419 (18.0) |
| MAAS | 910 | 110 | 12.1% | 913 | 78 | 8.5% | 1823 | 188 (9.8) |
| MYHAT | 1161 | 132 | 11.4% | 735 | 110 | 15.0% | 1896 | 242 (12.6) |
| SALSA | 999 | 58 | 5.8% | 711 | 31 | 4.4% | 1710 | 89 (5.0) |
| SAS | 2034 | 1134 | 55.8% | 1643 | 884 | 53.8% | 3677 | 2018 (52.6) |
| SGS | 1475 | 954 | 64.7% | 1142 | 731 | 64.0% | 2617 | 1685 (64.1) |
| SLAS II | 1992 | 1058 | 53.1% | 1192 | 686 | 57.6% | 3184 | 1744 (53.3) |
| SMAS | 572 | 79 | 13.8% | 465 | 51 | 11.0% | 1037 | 130 (12.5) |
| SPAH | 1187 | 203 | 17.1% | 780 | 166 | 21.3% | 1967 | 369 (17.8) |
| ZARADEMP | 2606 | 817 | 31.4% | 1974 | 526 | 26.6% | 4580 | 1343 (28.0) |
|  |  |  |  |  |  |  |  |  |
| Overall | 25032 | 7570 | 30.2% | 17993 | 5605 | 31.2% | 43025 | 13175 (30.6) |

Number at baseline excluded those with baseline dementia and without sex recorded.

**Supplementary Table 4: Baseline characteristics of participants excluded from 21 COSMIC cohorts due to no follow-up.**

|  | **Participants lost to follow-up (N=13,175)** | **Participants included in the analyses**  **(N=29,850)** |
| --- | --- | --- |
|  | **N, %** | **N, %** |
| Sex: |  |  |
| Men | 5605 (42.5) | 12555 (42.1) |
| Women | 7570 (57.5) | 17295 (57.9) |
| Baseline age: |  |  |
| <80 years | 10174 (77.2) | 24150 (80.9) |
| ≥80 years | 2765 (21.0) | 5451 (18.3) |
| Education years: |  |  |
| ≥9 years | 6630 (50.3) | 15142 (50.7) |
| <9 years | 6135 (46.6) | 14596 (48.9) |
| Country-level economies: |  |  |
| High income | 7698 (58.4) | 19116 (64.0) |
| Upper-middle income | 3738 (28.4) | 8753 (29.3) |
| Low to lower-middle income | 1517 (11.5) | 1981 (6.6) |
| Region: |  |  |
| Western countries | 2686 (20.4) | 11872 (39.8) |
| Asian countries | 6797 (51.6) | 8031 (26.9) |
| Other | 1518 (11.5) | 3477 (11.6) |
| *APOE* genotype: |  |  |
| *ε3/ε3* | 2102 (16.0) | 7936 (26.6) |
| *ε2* carriage | 444 (3.4) | 1667 (5.6) |
| *ε4* carriage | 658 (5.0) | 2745 (9.2) |
| Birth cohort: |  |  |
| After 1934 | 2328 (17.7) | 7141 (23.9) |
| 1925 to 1934 | 2613 (19.8) | 10148 (34.0) |
| Before 1925 | 5815 (44.1) | 12312 (41.2) |

**Supplementary Table 5: Definition of all-cause dementia in each cohort.**

| **Study** | **Dementia definition** |
| --- | --- |
| BCSA | MMSE score cut-off point 13 appropriate for Brazilian populations with low schooling (deemed appropriate for Brazilian populations with low schooling)^1^ |
| CLAS | Dementia was diagnosed according to the DSM-IV criteria. |
| EAS | Cognitive function, including performance on various neuropsychological tests measuring different cognitive domains was completed. Subjective memory impairment and functional decline were also assessed. If possible, informant reports of cognitive impairment and functional decline were obtained. Clinical diagnoses were determined according to DSM-IV criteria and assigned at consensus case conferences which included the study neurologist and the study neuropsychologist who comprehensively reviewed cognitive test results, relevant neurological signs and symptoms, and assessments of functional status. A similar procedure was adopted for all follow-up assessments. To ensure that diagnostic criteria were uniform over time, all individuals evaluated before the release of DSM-IV in 1994 were retrospectively reconferenced according to DSM-IV criteria. |
| EPIDEMCA | Cognitive disorders were assessed at baseline and follow-up assessments through a two-phase screening and diagnostic procedure. The Community Screening Interview for Dementia (CSI-D) was administered to identify suspected cases of dementia. All participants obtaining a poor performance on the CSI-D (COGSCORE ≤ 24.5) were referred for detailed clinical assessment with a neurologist. A neurological examination was performed. Orientation skills and daily living activities were also investigated to evaluate dependence. Neurological assessments also involved additional cognitive tests. Consensus diagnosis was determined according to DSM-IV criteria and based on reviewing of all medical records and performance on clinical assessments and cognitive tests. |
| ESPRIT | A neurologist examined all participants at baseline using a standardised clinical interview, as well as a battery of cognitive tests, to identify both dementia and mild cognitive impairment. A panel of expert neurologists further validated all incident cases of dementia independently from the study investigators. Diagnoses were determined according to DSM-IV criteria. The same procedure was followed for all follow-up assessments. |
| H70-study | Dementia was diagnosed according to DSM-III-R criteria, using information from neuropsychiatric examination, including a battery of cognitive tests, as well as a semi-structured close informant interview. The neuropsychiatric examinations and the close informant interviews were performed by medical doctors, psychologists, or psychiatric research nurses. At each assessment occasion, dementia status was determined based on computerized algorithms and a consensus conference including at least two psychiatrists. |
| HELIAD | Certified neurologists and trained neuropsychologists administered structured questionnaires and conducted neuropsychological assessments. Trained psychometricians administered a battery of neuropsychological tests to assess major cognitive domains. Information collected from all evaluations was reviewed at expert consensus meetings, including the neurologists who examined the participants clinically and neuropsychologists. Dementia diagnoses were made in accordance with DSM-IV criteria, using the same procedure at each assessment. |
| Invece.Ab | Clinical interview and visit are executed by expert geriatricians, members of the same geriatric staff who apply DSM-IV. Every diagnostic conclusion is revised by another doctor; in case of discrepancies a third geriatrician, chief of the study, intervenes to arbitrate. |
| ISA | At follow-up, a psychiatrist reviewed all available information to determine the presence or absence of dementia. The information included scores on the 10-WDRT and CHIF, the interviewer’s observations (of respondent’s memory and language, which the interviewers recorded at the end of each respondent’s assessment), reported functional status (as obtained in several sections of the interview and commonly supplemented by key informant’s report), and the temporal relationship of the onset of any co-occurring depressive disorder. Although the CHIF obtained information of respondent’s higher cognitive function by assessing their knowledge of how to perform instrumental ADLs, role functioning was evaluated to determine difficulties in the performance of ADLs and instrumental ADLs. The psychiatrist used all of the information in making a determination of presence or absence of dementia. |
| Leiden85+ | Dementia in the Leiden 85+ Study was defined as a clinical diagnosis based on the questionnaire filled in by the general practitioner of that participant. |
| LEILA75+ | At each assessment occasion, a fully structured interview was administered to the study subjects by trained physicians and psychologists. A battery of cognitive tests was administered. Additional information on cognitive and psychosocial functioning was obtained through fully structured informant interviews. Consensus conferences were held to determine dementia status in accordance with DSM-IV criteria. |
| LRGS TUA | Based on the Clinical Dementia Rating Scale (a CDR score of 1+ = dementia). |
| MAAS | At baseline, participants were excluded based on an existing diagnosis of dementia or a score below 24 on the Mini-Mental State Examination (MMSE). Included participants were assessed on a battery of cognitive tests, as well as activities of daily living and subjective cognitive complaints. A general practitioner and the investigators identified cases of dementia in accordance with DSM-III-R/DSM-IV criteria using all available clinical information and neuropsychological test data at all follow up occasions |
| MYHAT | At baseline, trained interviewers administered the MMSE (corrected for age and education). Individuals scoring less than 21 of 30 were classified as moderately or severely impaired and not assessed further. Included participants received detailed clinical and neuropsychological assessments at baseline and all follow-up assessments. Dementia was assessed through a purely cognitive classification and through assessment of everyday functioning (using the CDR). For cognitive classification, participants received a battery of tests assessing numerous cognitive domains. Composite scores for each cognitive domain were created. Normative reference points were created for each domain (excluding participants with CDR>1). On the basis of these reference points, participants were classified as dementia cases if composite scores in at least two domains were >2.0 SD below the mean for the individual's reference group. The CDR was administered by trained and certified interviewers and used to classify dementia cases based solely on measures of everyday functioning. CDR >1 was used to determine the presence of dementia and classification for each participant was finalized by consensus among 2 or more interviewers, ignoring the neuropsychological data but determining that the reported or observed functional impairments were attributable to cognitive difficulties. |
| SALSA | Cognitive screening tests were used to determine the need for further neuropsychological evaluation. Participants received formal neuropsychological evaluation if adjusted screening scores fell below the 20th percentile on any adjusted test. Formal neuropsychological evaluation included administration of a battery of cognitive tests, as well as the Informant Questionnaire on Cognitive Decline in the Elderly. Participants were referred for a neurological examination if they met at least one of the following criteria: (1) a score below the 10th per-centile on one or more of the six neuropsychological tests and a score on the IQCODE of 3.40 or greater (2) impaired scores on four or more neuropsychological tests regardless of IQCODE, or (3) IQCODE greater than 4.0. A team of neurologists and a neuropsychologist determined all dementia cases via consensus and in accordance with DSM-IV criteria at each assessment occasion. |
| SAS | After each clinical assessment, study neurologists and neuropsychologists reviewed the functional, medical, neurologic, psychiatric, and neuropsychological data and reached a consensus regarding the presence or absence of dementia using DSM-IV criteria. |
| SGS | Cognitive function tests (MoCA and MMSE). |
| SLAS II | Initial screening for cognitive impairment was performed using self or informant reports of subjective cognitive decline and the locally validated translated version of the MMSE, using a screening threshold of 26/27. Screened participants were further assessed using the CDR Scale. A battery of neurocognitive tests, previously validated with normative values for use in Singaporean adults, were used for assessment of cognitive domains, including memory [Rey Auditory Verbal Learning Test (RAVLT) immediate recall, RAVLT delayed recall, Visual Reproduction immediate recall, Visual Reproduction delayed]; executive function (Symbol Digit Modality Test, Design Fluency, Trail Making Test B); language (Categorical Verbal Fluency); visuospatial skills (Block Design); and attention (Digit Span Forwards, Digit Span Backwards, Spatial Span Forwards and Backwards). The final clinical assessments included magnetic resonance imaging and consensus diagnosis by a panel of geriatricians and psychiatrists. Dementia was diagnosed based on the Diagnostic and Statistical Manual of Mental Disorders (DSM), with evidence of cognitive deficit (MMSE ≤23 or neuropsychological domain scores <2 SD of age–education-adjusted mean) and evidence of functional impairment (dependency in ≥1 activities of daily living or CDR score ≥1). |
| SMAS | At baseline, participants were excluded if they had a previous diagnosis of dementia, if they had a MMSE score of < 24 (adjusted for age, education, and non-English speaking background at study entry), or if they received a diagnosis of dementia after comprehensive baseline assessments. A comprehensive neuropsychological battery was administered to assess numerous cognitive domains. Individuals who had: a score of at least 1.5 standard deviations below published normative data on a memory and a non-memory measure, or on two non-memory measures, or reduced neuropsychological scores and a decline in activities of daily living based on an informant interview were further assessed. Consensus diagnoses were made in accordance with DSM-IV criteria, by an expert team comprising neuropsychiatrists, psycho-geriatricians, and neuropsychologists, on the basis of the available clinical, neuropsychological, laboratorial and imaging data. A similar procedure was followed for each assessment occasion. |
| SPAH | The assessment of dementia was carried out with a harmonized one-phase dementia diagnostic procedure developed by the 10/66 Dementia Research Group and a detailed assessment of the onset and course of the dementia syndrome. This procedure included the Community Screening Instrument for Dementia (CSI-D), a battery of cognitive tests and a structured neurological assessment. An interview with the informants assessed participant’s cognitive and daily function and a brief history of the participant’s functional and cognitive decline. Diagnosis of dementia was made in accordance with DSM-IV criteria and based on all information collected from the subject and his/her informant. A similar procedure was followed for each assessment occasion. |
| ZARADEMP | A two-phase epidemiological case-finding process for dementia was implemented in the baseline study and a similar method in the follow up waves. In phase I, well-trained and regularly supervised lay interviewers conducted the ZARADEMP interview, including numerous cognitive tests and screening instruments. Medical reports and laboratory data were consulted to complete the data. Outside caregivers were interviewed when the participant was considered unreliable. Participants were considered probable cases based on standard cut-off points used for the Geriatric Mental State and/or the MMSE. In phase II, the probable cases of dementia were reassessed by research psychiatrists. The same assessment instruments and methods were used, and a neurological examination was performed. Identified cases of dementia were presented to a panel of four research psychiatrists for final diagnostic decisions, which were made in accordance with DSM-IV criteria. |

ADAS-COG, Alzheimer's Disease Assessment Scale–Cognitive Subscale; ADL, ‎Activities of daily living; CHIF, Clinician Home-based Interview to assess Function; CDR, Clinical Dementia Rating; COGSCORE, General Practitioner Assessment of Cognition Score; CSI-D, Community Screening Instrument for Dementia; DRS, Dementia Rating Scale; DSM, Diagnostic and Statistical Manual of Mental Disorders; MCI, mild cognitive impairment; MMSE, Mini–Mental State Examination; MoCA, Montreal Cognitive Assessment; RAVLT, Rey Auditory Verbal Learning Test; SD, standard deviation; SCID, Structured Clinical Interview for DSM Disorders; 10-WDRT, 10-Word Delayed Recall Test.

^1^ Castro-Costa E, Fuzikawa C, Uchoa E, Firmo JO, Lima-Costa MF. Norms for the mini-mental state examination: adjustment of the cut-off point in population-based studies (evidences from the Bambui health aging study). Arq Neuropsiquiatr 2008;66:524-8.

**Supplementary Table 6: Harmonisation for years assigned to educational attainment categories.**

| **Study** | **Years assigned to education attainment categories** |
| --- | --- |
| BCSA | Education attainment in years was recorded in original dataset. |
| CLAS | Illiterate = 0  Primary school = 6  Junior high school = 9  Senior high school = 12  Training school = 13  University or above = 15 |
| EAS | Education attainment in years was recorded in original dataset. |
| EPIDEMCA | Never attended school = 0  Schooled but never completed primary = 3  Primary completed = 6  Secondary completed = 12  Tertiary or higher education = 15 |
| ESPRIT | Below high school = 6  High school = 9  College = 12  University =15 |
| H70-study | Education attainment in years was recorded in original dataset. |
| HELIAD | Education attainment in years was recorded in original dataset. |
| Invece.Ab | Education attainment in years was recorded in original dataset. |
| ISA | No education = 0  Primary = 6  Secondary = 12  Tertiary **=** 15 |
| Leiden85+ | None = 0  Lower general education = 9  Lower vocational education = 9  Middle general education = 10  Middle vocational education = 10  General secondary education = 12  Higher vocational education = 15  University = 16  Other = NA  Unknown = NA |
| LEILA75+ | Education attainment in years was recorded in original dataset. |
| LRGS TUA | Education attainment in years was recorded in original dataset. |
| MAAS | Elementary education = 6  Lower vocational education = 9  Intermediate secondary education = 9  Intermediate vocational education = 10  Higher secondary education = 11  Higher vocational education = 15  University education = 16  Scientific education = 17 |
| MYHAT | Education attainment in years was recorded in original dataset. |
| SALSA | Education attainment in years was recorded in original dataset. |
| SAS | Education attainment in years was recorded in original dataset. |
| SGS | Education attainment in years was recorded in original dataset. |
| SLAS II | Education attainment in years was recorded in original dataset. |
| SMAS | Education attainment in years was recorded in original dataset. |
| SPAH | Education attainment in years was recorded in original dataset.  None = 0  Some, did not complete primary = 3  Completed primary = 6  Completed secondary = 12  Tertiary (college) = 15 |
| ZARADEMP | Education attainment in years was recorded in original dataset. |

**Supplementary Table 7: Harmonisation protocol for hypertension.**

| **Study** | **Criteria for hypertension (meeting any of the criteria was sufficient)** |
| --- | --- |
| BCSA | Systolic and diastolic blood pressure  Received treatments for hypertension  Previous medical diagnosis |
| CLAS | Systolic and diastolic blood pressure  Received treatments for hypertension  Self-reported hypertension |
| EAS | Systolic and diastolic blood pressure  Received treatments for hypertension |
| EPIDEMCA | Systolic and diastolic blood pressure  Received treatments for hypertension  Self-reported hypertension |
| ESPRIT | Systolic and diastolic blood pressure  Received treatments for hypertension  Self-reported hypertension |
| H70-study | Systolic and diastolic blood pressure  Received treatments for hypertension  Self-reported hypertension |
| HELIAD | Systolic and diastolic blood pressure  Received treatments for hypertension  Self-reported hypertension |
| Invece.Ab | Systolic and diastolic blood pressure  Received treatments for hypertension |
| ISA | **-** |
| Leiden85+ | Systolic and diastolic blood pressure  Received treatments for hypertension |
| LEILA75+ | Systolic and diastolic blood pressure |
| LRGS TUA | Systolic and diastolic blood pressure  Received treatments for hypertension  Self-reported hypertension  Previous medical diagnosis |
| MAAS | Systolic and diastolic blood pressure  Previous medical diagnosis: essential hypertension or hypertension with known aetiology  Received treatments for hypertension |
| MYHAT | Systolic and diastolic blood pressure  Received treatments for hypertension |
| SALSA | Systolic and diastolic blood pressure  Received treatments for hypertension |
| SAS | Systolic and diastolic blood pressure  Received treatments for hypertension |
| SGS | Systolic and diastolic blood pressure |
| SLAS II | Systolic and diastolic blood pressure  Received treatments for hypertension |
| SMAS | Systolic and diastolic blood pressure  Received treatments for hypertension |
| SPAH | Systolic and diastolic blood pressure  Received treatments for hypertension |
| ZARADEMP | Systolic and diastolic blood pressure  Received treatments for hypertension |

Systolic blood pressure ≥140mmHg and/or diastolic blood pressure≥90mmHg.

**Supplementary Table 8: Harmonisation protocol for diabetes mellitus.**

| **Study** | **Criteria for diabetes (meeting any of the criteria was sufficient)** |
| --- | --- |
| BCSA | Fasting blood glucose  Current use of insulin or oral antidiabetic drug treatment |
| CLAS | Fasting blood glucose  Received treatments  Self-reported diabetes |
| EAS | Received treatments  Told to have diabetes |
| EPIDEMCA | Received treatments  Fasting blood glucose |
| ESPRIT | Fasting blood glucose  Received treatments  Self-reported diabetes |
| H70-study | Fasting blood glucose  Received treatments  Self-reported diabetes |
| HELIAD | Blood glucose  Received treatments  Self-reported diabetes |
| Invece.Ab | Fasting blood glucose  Received treatments  Self-reported diabetes |
| ISA | Self-reported diabetes |
| Leiden85+ | Fasting blood glucose  Received treatments  Known diabetes to doctors or pharmacists |
| LEILA75+ | Received treatments  Self-reported diabetes |
| LRGS TUA | Fasting blood glucose  Received treatments  Self-reported diabetes |
| MAAS | Received treatments age 40+  Self-reported diabetes |
| MYHAT | Fasting blood glucose  Received treatments  Self-reported diabetes |
| SALSA | Self-reported  Fasting blood glucose  Received treatments |
| SAS | Fasting blood glucose  Received treatments  Self-reported diabetes |
| SGS | Self-reported diabetes |
| SLAS II | Fasting blood glucose  Received treatments  Self-reported diabetes |
| SMAS | Fasting blood glucose  Received treatments  Diabetes confirmed by doctors |
| SPAH | Fasting blood glucose  Received treatments |
| ZARADEMP | Diagnosis using EURODEM Risk Factor Questionnaire and medical records |

EURODEM, European Community Concerted Action Epidemiology of Dementia.

Fasting blood glucose criteria are ≥126mg/dL or >7mmol/L.

**Supplementary Table 9: Harmonisation protocol for depression.**

| **Study** | **Criteria for current or a history of depression (meeting any of the criteria was sufficient)** |
| --- | --- |
| BCSA | General Health Questionnaire-12 score 5+ |
| CLAS | GDS score 6+  History of depression yes/no |
| EAS | GDS score 6+ |
| EPIDEMCA | GMS score 3+ |
| ESPRIT | Current major depressive episode (MINI neuropsychiatric exam)  CES-D score 16+  History of major depressive episode (MINI neuropsychiatric exam)  Current antidepressant use |
| H70-study | MADRS score 18+  Self-reported minor or major depression  Current antidepressant use |
| HELIAD | GDS score 6+ |
| Invece.Ab | GDS score 6+  Criteria-based diagnosis by physician/psychologist (including medication, GDS score and CES-D items)  History of depression  Current antidepressant use |
| ISA | GDS score 6+  Self-reported 12 months depressive symptoms |
| Leiden85+ | GDS score 6+  Current antidepressant use |
| LEILA75+ | DSM-IV criteria based on structured clinical interview  CES-D score 16+  Self-reported history of depression |
| LRGS TUA | GDS score 6+ |
| MAAS | Current antidepressant use |
| MYHAT | >90% percentile on modified CES-D  Self-reported history of depression  Current antidepressant use |
| SALSA | CES-D score 16+  Use of anti-depressants |
| SAS | CES-D score 16+  Use of anti-depressants |
| SGS | K6 score 5+  Self-reported history of diagnosis |
| SLAS II | GDS score 6+  Current antidepressant use  Self-reported history of diagnosis |
| SMAS | GDS score 6+  Current antidepressant use  Self-reported history of diagnosis |
| SPAH | Current antidepressant use  Depression based on ICD-10 |
| ZARADEMP | GMS-AGECAT rating of subcase or clinical case  Depression or antidepressant use or ever treated by primary care, psychiatric or hospitalised |

CES-D, Centre for Epidemiological Studies depression scale; DSM-IV, Diagnostic and Statistical Manual of Mental Disorders (4th edition); GDS, Geriatric Depression Scale. GMS-AGECAT, Geriatric Mental State-Automated Geriatric Examination for Computer Assisted Taxonomy; ICD-10, International Classification of Diseases (10th revision); K6, Kessler Psychological Distress Scale; MADRS, Montgomery-Asberg Depression Rating Scale; MINI, Mini International Neuropsychiatric Interview.

**Supplementary Table 10: Harmonisation protocol for hearing impairment.**

| **Study** | **Criteria for hearing impairment (meeting any of the criteria was sufficient)** |
| --- | --- |
| BCSA | - |
| CLAS | - |
| EAS | - |
| EPIDEMCA | Severe deafness |
| ESPRIT | - |
| H70-study | Self-reported hearing loss |
| HELIAD | Interviewer's judgment |
| Invece.Ab | Self-perceived hearing loss  Clinician-evaluated hearing loss (Whispered Voice Test) |
| ISA | Interviewer’s judgement |
| Leiden85+ | Self-reported hearing loss |
| LEILA75+ | Self-report hearing impairment |
| LRGS TUA | - |
| MAAS | Can you hear well (potentially with a hearing aid) yes/no? |
| MYHAT | Self-reported hearing loss |
| SALSA | - |
| SAS | - |
| SGS | - |
| SLAS II | - |
| SMAS | Self-reported hearing loss |
| SPAH | Severe deafness |
| ZARADEMP | **-** |

**Supplementary Table 11: Harmonisation protocol for alcohol use.**

| **Study** | **Criteria for alcohol use** |
| --- | --- |
| BCSA | Current drinker: Positive answer to “Drink at least once a week” |
| CLAS | Current drinker: Have drank in the past and have not withdrawn from drinking  Former drinker: Have drank in the past and have withdrawn from drinking |
| EAS | Current drinker: Positive answer to “Have you had at least one drink of beer, wine or liquor during the past year?” |
| EPIDEMCA | Current drinker: Reported drinking regularly (at least 5 days/week) or sometimes (can stop anytime) |
| ESPRIT | Current drinker: Drinks per week calculated from consumption in grams/day using 10 grams ≥ 1  Former drinker: Formerly drank alcohol and not currently drinking |
| H70-study | Current drinker: Estimated total alcohol consumption(gram/week) ≥ 1 gram |
| HELIAD | Current drinker: Drinks per week calculated from frequency and glasses consumed per occasion for each of tsipouro, beer, wine, whisky  Former drinker: Formerly drank alcohol and not currently drinking |
| Invece.Ab | Current drinker: Currently drinking alcohol |
| ISA | - |
| Leiden85+ | Current drinker: ≥ 1 unit alcohol per week |
| LEILA75+ | Current drinker: ≥ 1 unit alcohol per week  Former drinker: Formerly drank alcohol and not currently drinking |
| LRGS TUA | Current drinker: positive answer to “Do you take alcohol?” |
| MAAS | Current drinker: If reported ≥ 1 for the question “If you drink alcohol: how many drinks on average per day?” |
| MYHAT | Current drinker: Currently drinking alcohol  Former drinker: Formerly drank alcohol and not currently drinking |
| SALSA | Current drinker: Any alcohol consumption and/or number of drinks per week ≥ 1 unit |
| SAS | Current drinker: Currently drinking alcohol and/or number of drinks per week ≥ 1 unit |
| SGS | Current drinker: Drinking almost every day or drinking sometimes |
| SLAS II | Current drinker: Drinking beer, wine, or liquor or other alcoholic drinks more than once a month |
| SMAS | Current drinker: Have drank alcohol and number of drinks per week ≥ 1  Former drinker: Have drank alcohol and number of drinks per week = 0 |
| SPAH | Current drinker: Number of drinks per week ≥ 1 unit |
| ZARADEMP | Current drinker: Currently drinking alcohol  Former drinker: Formerly drank alcohol and not currently drinking |

**Supplementary Table 12: Harmonisation protocol for physical activity.**

| **Study** | **Criteria for physical activity based on WHO recommendation** * **(meeting any of the criteria was sufficient, otherwise classified as none to minimal physical activity)** |
| --- | --- |
| BCSA | Any positive answer determined by the response to the question “During leisure times in the previous 90 days, how many times did you walk to exercise, do gymnastics or practice sports for at least 20 to 30 min?” |
| CLAS | Practice any sports or Tai Chi per exercise for more than 20 minutes |
| EAS | Light exercise four or more days per week, or 10 or more hours per two weeks  Medium exercise three or more days per week, or 5 or more hours per two weeks  Heavy exercise two or more days per week, or 2.5 or more hours per two weeks |
| EPIDEMCA | Physical activity ≥ 150 minutes per week |
| ESPRIT | Sports activity frequency: regular or often |
| H70-study | Walking 3 to 4 days per week or almost daily  Walking duration longer than 30 minutes each time  Light physical activity 2 to 4 hours a week per week in summer |
| HELIAD | Dance, walk or doing other types of exercise ≥ 2.5 hours per week |
| Invece.Ab | Walking ≥ 30 minutes per day  Reported doing one of the following exercises: biking, dancing, gymnastic, swimming, running, tennis, and aerobics |
| ISA | Moderate to high physical activity |
| Leiden85+ | **-** |
| LEILA75+ | **-** |
| LRGS TUA | Reported doing often to very often to the following questions: “Do you drive or cycle or ride bike?” or “Are you involved in exercises such as aerobic, walking, jogging” or very often to the question: “Are you involved in recreational activities such as golf, bowling, or kite flying” |
| MAAS | ≥ 2.5 hours to the question “How many hours per week spent on sports: ball sports, fitness training, etc.?”  ≥ 5 hours to the question “How many hours per week spent on light sports: walking, cycling, gardening etc.?” |
| MYHAT | Exercised in the past year |
| SALSA | ≥ 35 METs hours per week |
| SAS | ≥ 35 METs hours per week |
| SGS | Time spent in moderate to vigorous physical activity in minutes per day (intensity ≥3 METs) |
| SLAS II | Light activity, usual weekday(hours/day) = 5 per week  Medium activity, usual weekday(hours/day) = 2.5 per week  Heavy activity, usual weekday (hours/day) = 1.25 per week |
| SMAS | Reported doing one of the following sports: bowling, dancing, walking, tennis, swimming, jogging, bicycling, aerobics and other sports |
| SPAH | Positive answer to the question “Taking into account both work and leisure, would you say that you are very or reasonably active?” |
| ZARADEMP | **-** |

MET, metabolic equivalent of task.

* WHO recommendation: Adults aged 65 years and above: Should do at least 150 minutes of moderate-intensity physical activity throughout the week, or at least 75 minutes of vigorous-intensity physical activity, or an equivalent combination of both (Source: https://www.who.int/news-room/fact-sheets/detail/physical-activity).

**Supplementary Table 13: Harmonisation protocol for high cholesterol.**

| **Study** | **Criteria for high cholesterol (meeting any of the criteria was sufficient)** |
| --- | --- |
| BCSA | - |
| CLAS | Total cholesterol  Receive lipid-lowering medications  Self-reported high cholesterol |
| EAS | Total cholesterol |
| EPIDEMCA | Total cholesterol |
| ESPRIT | Total cholesterol  Receive lipid-lowering medications  Self-reported high cholesterol |
| H70-study | Total cholesterol  Receive lipid-lowering medications |
| HELIAD | Total cholesterol  Receive lipid-lowering medications  Self-reported high cholesterol |
| Invece.Ab | Total cholesterol |
| ISA | **-** |
| Leiden85+ | Total cholesterol  Receive lipid-lowering medications |
| LEILA75+ | - |
| LRGS TUA | Total cholesterol  Receive lipid-lowering medications  Self-reported high cholesterol  High cholesterol diagnosed |
| MAAS | Receive lipid-lowering medications |
| MYHAT | Total cholesterol  Receive lipid-lowering medications |
| SALSA | Total cholesterol  Receive lipid-lowering medications |
| SAS | Total cholesterol  Receive lipid-lowering medications |
| SGS | Self-reported high cholesterol |
| SLAS II | Total cholesterol  Receive lipid-lowering medications |
| SMAS | Total cholesterol  Receive lipid-lowering medications |
| SPAH | Total cholesterol  Receive lipid-lowering medications |
| ZARADEMP | Self-reported high cholesterol |

Total cholesterol ≥6.2 mmol/L.

**Supplementary Table 14: Missing data for the 21 contributing COSMIC cohorts, by sex.**

|  | **BCSA** | | **CLAS** | | **EAS** | | **EPIDEMCA** | | **ESPRIT** | | **H-70 Study** | | **HELIAD** | |
| --- | --- | --- | --- | --- | --- | --- | --- | --- | --- | --- | --- | --- | --- | --- |
|  | **Women** | **Men** | **Women** | **Men** | **Women** | **Men** | **Women** | **Men** | **Women** | **Men** | **Women** | **Men** | **Women** | **Men** |
| Age (N, %) | 0 (0.0) | 0 (0.0) | 132 (11.7) | 110 (11.4) | 0 (0.0) | 0 (0.0) | 0 (0.0) | 0 (0.0) | 0 (0.0) | 0 (0.0) | 0 (0.0) | 0 (0.0) | 2 (0.3) | 5 (1.2) |
| Education years (N, %) | 4 (0.3) | 2 (0.4) | 4 (0.4) | 3 (0.3) | 0 (0.0) | 0 (0.0) | 1 (0.3) | 1 (0.2) | 0 (0.0) | 1 (0.1) | 33 (6.0) | 1 (0.6) | 0 (0.0) | 0 (0.0) |
| Systolic blood pressure (N, %) | 46 (5.0) | 41 (7.0) | 21 (1.9) | 25 (2.6) | 465 (55.4) | 319 (60.8) | 4 (0.9) | 7 (2.2) | 98 (8.4) | 70 (8.6) | 71 (39.4) | 1 (0.1) | 27 (4.4) | 19 (4.7) |
| Diastolic blood pressure (N, %) | 46 (5.0) | 41 (7.0) | 23 (2.0) | 27 (2.8) | 465 (55.4) | 319 (60.8) | 4 (0.9) | 7 (2.2) | 98 (8.4) | 70 (8.6) | 100 (18.2) | 4 (2.2) | 27 (4.4) | 20 (4.9) |
| BMI (N, %) | 65 (5.6) | 45 (5.5) | 408 (36.0) | 316 (32.8) | 466 (55.5) | 318 (60.6) | 19 (4.4) | 15 (4.9) | 10 (0.9) | 2 (0.2) | 28 (5.1) | 1 (0.6) | 10 (1.7) | 6 (1.5) |
| Waist circumference (N, %) | 64 (5.5) | 45 (5.5) | 1133 (100.0) | 964 (100.0) | 465 (55.5) | 318 (60.6) | 28 (3.3) | 22 (4.2) | 1162 (100.0) | 815 (100.0) | 19 (3.4) | 2 (1.1) | 33 (5.5) | 20 (4.9) |
| Hip circumference (N, %) | 64 (5.5) | 45 (5.5) | 1133 (100.0) | 964 (100.0) | 466 (55.4) | 319 (60.8) | 26 (3.1) | 22 (4.2) | 1162 (100.0) | 815 (100.0) | 18 (3.3) | 2 (1.1) | 602 (100.0) | 406 (100.0) |
| Total cholesterol (N, %) | 912 (100.0) | 584 (100.0) | 730 (64.4) | 652 (67.6) | 503 (60.0) | 335 (63.8) | 76 (8.9) | 53 (10.0) | 17 (1.5) | 7 (0.9) | 15 (2.7) | 1 (0.6) | 212 (35.2) | 128 (31.5) |
| HDL cholesterol (N, %) | 44 (3.8) | 41 (5.0) | 732 (64.6) | 656 (68.0) | 503 (60.0) | 335 (63.8) | 429 (100.0) | 308 (100.0) | 17 (1.5) | 7 (0.9) | 15 (2.7) | 1 (0.6) | 286 (47.5) | 202 (49.8) |
| LDL cholesterol (N, %) | 912 (100.0) | 584 (100.0) | 733 (64.7) | 656 (68.0) | 503 (60.0) | 335 (63.8) | 429 (100.0) | 308 (100.0) | 20 (1.7) | 14 (1.7) | 20 (3.6) | 3 (1.7) | 289 (48.0) | 203 (50.0) |
| Triglycerides (N, %) | 44 (3.8) | 41 (5.0) | 730 (64.4) | 652 (67.6) | 503 (60.0) | 335 (63.8) | 429 (100.0) | 308 (100.0) | 17 (1.5) | 7 (0.9) | 551 (100.0) | 180 (100.0) | 212 (35.2) | 128 (31.5) |
| Diabetes (N, %) | 50 (4.3) | 52 (6.4) | 94 (8.3) | 92 (9.5) | 7 (0.8) | 4 (0.8) | 8 (1.9) | 6 (1.9) | 0 (0.0) | 0 (0.0) | 0 (0.0) | 0 (0.0) | 4 (0.7) | 2 (0.5) |
| Depression (N, %) | 7 (0.6) | 14 (1.7) | 10 (0.9) | 15 (1.6) | 129 (15.4) | 92 (17.5) | 0 (0.0) | 0 (0.0) | 58 (5.0) | 55 (6.7) | 0 (0.0) | 0 (0.0) | 0 (0.0) | 0 (0.0) |
| Hearing loss (N, %) | 912 (100.0) | 584 (100.0) | 1133 (100.0) | 964 (100.0) | 840 (100.0) | 525 (100.0) | 3 (0.7) | 0 (0.0) | 1162 (100.0) | 815 (100.0) | 32 (5.8) | 4 (2.2) | 3 (0.5) | 3 (0.7) |
| Current smoker (N, %) | 0 (0.0) | 0 (0.0) | 0 (0.0) | 0 (0.0) | 422 (50.2) | 188 (35.8) | 4 (0.9) | 1 (0.3) | 1 (0.1) | 0 (0.0) | 164 (29.8) | 1 (0.6) | 10 (1.7) | 6 (1.5) |
| Current alcohol use (N, %) | 5 (0.4) | 13 (1.6) | 0 (0.0) | 0 (0.0) | 6 (0.7) | 1 (0.2) | 1 (0.2) | 3 (1.0) | 4 (0.3) | 1 (0.1) | 40 (7.3) | 9 (5.0) | 10 (1.7) | 6 (1.5) |
| High physical activity (N, %) | 7 (0.6) | 4 (0.5) | 450 (39.7) | 309 (32.1) | 4 (0.5) | 1 (0.2) | 125 (29.1) | 89 (28.9) | 323 (27.8) | 17 (2.1) | 0 (0.0) | 0 (0.0) | 0 (0.0) | 1 (0.2) |
| APOE *ε*4 carrier (N, %) | 101 (8.7) | 67 (8.2) | 1133 (100.0) | 964 (100.0) | 339 (40.4) | 193 (36.8) | 134 (31.2) | 102 (33.1) | 23 (2.0) | 9 (1.1) | 7 (1.3) | 0 (0.0) | 195 (32.4) | 102 (25.1) |
|  | **Invece.Ab** | | **ISA** | | **Leiden85+** | | **LEILA75+** | | **LRGS TUA** | | **MAAS** | | **MYHAT** | |
|  | **Women** | **Men** | **Women** | **Men** | **Women** | **Men** | **Women** | **Men** | **Women** | **Men** | **Women** | **Men** | **Women** | **Men** |
| Age (N, %) | 0 (0.0) | 0 (0.0) | 0 (0.0) | 0 (0.0) | 0 (0.0) | 0 (0.0) | 0 (0.0) | 0 (0.0) | 0 (0.0) | 0 (0.0) | 0 (0.0) | 0 (0.0) | 0 (0.0) | 0 (0.0) |
| Education years (N, %) | 0 (0.0) | 0 (0.0) | 0 (0.0) | 0 (0.0) | 2 (0.6) | 3 (1.8) | 20 (2.8) | 4 (1.6) | 0 (0.0) | 0 (0.0) | 0 (0.0) | 1 (0.1) | 0 (0.0) | 0 (0.0) |
| Systolic blood pressure (N, %) | 8 (1.3) | 3 (0.6) | 639 (100.0) | 605 (100.0) | 2 (0.6) | 5 (3.0) | 57 (8.0) | 10 (4.0) | 55 (5.7) | 39 (4.2) | 20 (2.5) | 14 (1.7) | 9 (0.9) | 1 (0.2) |
| Diastolic blood pressure (N, %) | 8 (1.3) | 3 (0.6) | 639 (100.0) | 605 (100.0) | 2 (0.6) | 5 (3.0) | 57 (8.0) | 10 (4.0) | 55 (5.7) | 39 (4.2) | 21 (2.6) | 14 (1.7) | 10 (1.0) | 1 (0.2) |
| BMI (N, %) | 69 (11.3) | 55 (10.6) | 639 (100.0) | 605 (100.0) | 20 (3.3) | 8 (1.3) | 712 (100.0) | 250 (100.0) | 2 (0.2) | 5 (0.5) | 2 (0.3) | 0 (0.0) | 15 (1.5) | 15 (2.4) |
| Waist circumference (N, %) | 32 (5.3) | 16 (3.1) | 639 (100.0) | 605 (100.0) | 350 (100.0) | 169 (100.0) | 712 (100.0) | 250 (100.0) | 3 (0.3) | 6 (0.6) | 5 (0.6) | 1 (0.1) | 61 (5.9) | 24 (3.8) |
| Hip circumference (N, %) | 608 (100.0) | 519 (100.0) | 639 (100.0) | 605 (100.0) | 350 (100.0) | 169 (100.0) | 712 (100.0) | 250 (100.0) | 3 (0.3) | 6 (0.6) | 5 (0.6) | 3 (0.4) | 62 (6.0) | 24 (3.8) |
| Total cholesterol (N, %) | 7 (1.2) | 6 (1.2) | 639 (100.0) | 605 (100.0) | 8 (1.3) | 5 (0.8) | 712 (100.0) | 250 (100.0) | 56 (5.8) | 62 (6.7) | 793 (100.0) | 842 (100.0) | 491 (47.7) | 275 (44.0) |
| HDL cholesterol (N, %) | 7 (1.2) | 6 (1.2) | 639 (100.0) | 605 (100.0) | 8 (1.3) | 5 (0.8) | 712 (100.0) | 250 (100.0) | 236 (24.3) | 165 (17.7) | 793 (100.0) | 842 (100.0) | 491 (47.7) | 275 (44.0) |
| LDL cholesterol (N, %) | 7 (1.2) | 6 (1.2) | 639 (100.0) | 605 (100.0) | 11 (1.8) | 6 (0.9) | 712 (100.0) | 250 (100.0) | 240 (24.7) | 167 (17.9) | 793 (100.0) | 842 (100.0) | 1029 (100.0) | 625 (100.0) |
| Triglycerides (N, %) | 7 (1.2) | 6 (1.2) | 639 (100.0) | 605 (100.0) | 8 (1.3) | 5 (0.8) | 712 (100.0) | 250 (100.0) | 237 (24.4) | 170 (18.3) | 793 (100.0) | 842 (100.0) | 1029 (100.0) | 625 (100.0) |
| Diabetes (N, %) | 0 (0.0) | 0 (0.0) | 4 (0.6) | 0 (0.0) | 0 (0.0) | 0 (0.0) | 1 (0.1) | 2 (0.8) | 0 (0.0) | 0 (0.0) | 0 (0.0) | 0 (0.0) | 1 (0.1) | 0 (0.0) |
| Depression (N, %) | 0 (0.0) | 0 (0.0) | 0 (0.0) | 0 (0.0) | 59 (9.8) | 12 (1.9) | 0 (0.0) | 0 (0.0) | 14 (1.4) | 22 (2.4) | 0 (0.0) | 0 (0.0) | 1 (0.1) | 0 (0.0) |
| Hearing loss (N, %) | 0 (0.0) | 0 (0.0) | 43 (6.7) | 36 (6.0) | 0 (0.0) | 0 (0.0) | 0 (0.0) | 2 (0.8) | 972 (100.0) | 931 (100.0) | 2 (0.3) | 3 (0.4) | 0 (0.0) | 1 (0.2) |
| Current smoker (N, %) | 0 (0.0) | 0 (0.0) | 639 (100.0) | 605 (100.0) | 6 (1.0) | 2 (0.3) | 33 (4.6) | 31 (12.4) | 0 (0.0) | 0 (0.0) | 187 (23.6) | 244 (29.0) | 366 (35.6) | 386 (61.8) |
| Current alcohol use (N, %) | 0 (0.0) | 1 (0.2) | 639 (100.0) | 605 (100.0) | 2 (0.3) | 1 (0.2) | 712 (100.0) | 250 (100.0) | 0 (0.0) | 0 (0.0) | 0 (0.0) | 0 (0.0) | 1 (0.1) | 0 (0.0) |
| High physical activity (N, %) | 0 (0.0) | 1 (0.2) | 0 (0.0) | 0 (0.0) | 350 (100.0) | 169 (100.0) | 712 (100.0) | 250 (100.0) | 15 (1.5) | 13 (1.4) | 92 (11.6) | 69 (8.2) | 1 (0.1) | 0 (0.0) |
| APOE *ε*4 carrier (N, %) | 0 (0.0) | 1 (0.2) | 639 (100.0) | 605 (100.0) | 14 (2.3) | 11 (1.7) | 516 (72.5) | 201 (80.4) | 972 (100.0) | 931 (100.0) | 353 (44.5) | 343 (40.7) | 83 (8.1) | 36 (5.8) |
|  | **SALSA** | | **SAS** | | **SGS** | | **SLAS II** | | **SMAS** | | **SPAH** | | **ZARADEMP** | |
|  | **Women** | **Men** | **Women** | **Men** | **Women** | **Men** | **Women** | **Men** | **Women** | **Men** | **Women** | **Men** | **Women** | **Men** |
| Age (N, %) | 0 (0.0) | 0 (0.0) | 0 (0.0) | 0 (0.0) | 0 (0.0) | 0 (0.0) | 0 (0.0) | 0 (0.0) | 0 (0.0) | 0 (0.0) | 0 (0.0) | 0 (0.0) | 0 (0.0) | 0 (0.0) |
| Education years (N, %) | 0 (0.0) | 0 (0.0) | 0 (0.0) | 0 (0.0) | 4 (0.8) | 3 (0.7) | 2 (0.2) | 0 (0.0) | 0 (0.0) | 0 (0.0) | 0 (0.0) | 0 (0.0) | 10 (0.6) | 13 (0.9) |
| Systolic blood pressure (N, %) | 34 (3.6) | 36 (5.3) | 1 (0.1) | 3 (0.4) | 57 (10.9) | 62 (15.1) | 24 (2.6) | 8 (1.6) | 15 (3.0) | 7 (1.7) | 34 (3.5) | 16 (2.6) | 125 (7.0) | 122 (8.4) |
| Diastolic blood pressure (N, %) | 35 (3.7) | 37 (5.4) | 1 (0.1) | 3 (0.4) | 57 (10.9) | 62 (15.1) | 24 (2.6) | 8 (1.6) | 15 (3.0) | 7 (1.7) | 34 (3.5) | 16 (2.6) | 130 (7.3) | 124 (8.6) |
| BMI (N, %) | 49 (5.2) | 37 (5.4) | 2 (0.2) | 2 (0.3) | 55 (10.6) | 62 (15.1) | 26 (2.8) | 8 (1.6) | 8 (1.6) | 7 (1.7) | 36 (3.7) | 15 (2.4) | 5 (0.3) | 1 (0.1) |
| Waist circumference (N, %) | 37 (3.9) | 40 (5.9) | 900 (100.0) | 759 (100.0) | 521 (100.0) | 411 (100.0) | 25 (2.7) | 10 (2.0) | 3 (0.6) | 5 (1.2) | 984 (100.0) | 614 (100.0) | 1789 (100.0) | 1448 (100.0) |
| Hip circumference (N, %) | 941 (100.0) | 680 (100.0) | 900 (100.0) | 759 (100.0) | 521 (100.0) | 411 (100.0) | 26 (2.8) | 13 (2.6) | 17 (3.4) | 14 (3.4) | 984 (100.0) | 614 (100.0) | 1789 (100.0) | 1448 (100.0) |
| Total cholesterol (N, %) | 40 (4.3) | 41 (6.0) | 73 (8.1) | 30 (4.0) | 521 (100.0) | 411 (100.0) | 60 (6.4) | 31 (6.1) | 42 (8.5) | 18 (4.3) | 38 (3.9) | 31 (5.0) | 1789 (100.0) | 1448 (100.0) |
| HDL cholesterol (N, %) | 26 (2.8) | 25 (3.7) | 74 (8.2) | 30 (4.0) | 521 (100.0) | 411 (100.0) | 58 (6.2) | 26 (5.1) | 43 (8.7) | 19 (4.6) | 38 (3.9) | 31 (5.0) | 1789 (100.0) | 1448 (100.0) |
| LDL cholesterol (N, %) | 26 (2.8) | 29 (4.3) | 74 (8.2) | 30 (4.0) | 521 (100.0) | 411 (100.0) | 64 (6.9) | 35 (6.9) | 44 (8.9) | 21 (5.1) | 49 (5.0) | 39 (6.4) | 1789 (100.0) | 1448 (100.0) |
| Triglycerides (N, %) | 42 (4.5) | 44 (6.5) | 73 (8.1) | 30 (4.0) | 521 (100.0) | 411 (100.0) | 60 (6.4) | 32 (6.3) | 42 (8.5) | 18 (4.3) | 630 (64.0) | 390 (63.5) | 1789 (100.0) | 1448 (100.0) |
| Diabetes (N, %) | 22 (2.3) | 13 (1.9) | 0 (0.0) | 0 (0.0) | 0 (0.0) | 1 (0.2) | 0 (0.0) | 0 (0.0) | 0 (0.0) | 0 (0.0) | 0 (0.0) | 0 (0.0) | 14 (0.8) | 13 (0.9) |
| Depression (N, %) | 0 (0.0) | 0 (0.0) | 0 (0.0) | 0 (0.0) | 0 (0.0) | 0 (0.0) | 0 (0.0) | 0 (0.0) | 0 (0.0) | 0 (0.0) | 0 (0.0) | 0 (0.0) | 88 (4.9) | 43 (3.0) |
| Hearing loss (N, %) | 941 (100.0) | 680 (100.0) | 900 (100.0) | 759 (100.0) | 521 (100.0) | 411 (100.0) | 934 (100.0) | 506 (100.0) | 1 (0.2) | 0 (0.0) | 0 (0.0) | 0 (0.0) | 1789 (100.0) | 1448 (100.0) |
| Current smoker (N, %) | 221 (23.5) | 154 (22.6) | 0 (0.0) | 0 (0.0) | 2 (0.4) | 2 (0.5) | 4 (0.4) | 2 (0.4) | 3 (0.6) | 4 (1.0) | 5 (0.5) | 3 (0.5) | 0 (0.0) | 2 (0.1) |
| Current alcohol use (N, %) | 0 (0.0) | 0 (0.0) | 0 (0.0) | 0 (0.0) | 1 (0.2) | 2 (0.5) | 2 (0.2) | 3 (0.6) | 1 (0.2) | 0 (0.0) | 41 (4.2) | 11 (1.8) | 0 (0.0) | 3 (0.2) |
| High physical activity (N, %) | 941 (100.0) | 680 (100.0) | 2 (0.2) | 9 (1.2) | 72 (13.8) | 87 (21.2) | 32 (3.4) | 14 (2.8) | 0 (0.0) | 0 (0.0) | 3 (0.3) | 3 (0.5) | 1789 (100.0) | 1448 (100.0) |
| APOE *ε*4 carrier (N, %) | 941 (100.0) | 680 (100.0) | 900 (100.0) | 759 (100.0) | 521 (100.0) | 411 (100.0) | 51 (5.5) | 104 (20.6) | 5 (1.0) | 10 (2.4) | 131 (13.3) | 83 (13.5) | 1789 (100.0) | 1448 (100.0) |

**Supplementary Table 15: Ethics approval for the 21 contributing COSMIC cohorts.**

| **Study** | **Institutional Review Board** |
| --- | --- |
| BCSA | Ethics Boards of the Fundação Oswaldo Cruz in Rio de Janeiro and the Instituto Rene´ Rachou of the Fundação Oswaldo Cruz in Belo Horizonte, Brazil (14/2007 - CEPSH-CpqRR) |
| CLAS | Ethics Committee of Shanghai Mental Health Center (2011-YJ-14) |
| EAS | Albert Einstein College of Medicine Institutional Review Board (Approval#1996-175) |
| EPIDEMCA | Ethical committees, supervised by Ministry of Public Health in CAR (8/UB/FACSS/CSCVPER/11) and the Comité d’Ethique de la Recherche en Sciences de Santé in ROC (00000204/DGRST/CERSSA), approved the study protocol, as well as the “Comité de la Protection des Personnes Sud-Ouest Outre-Mer” in France (SOOM4/CE/3). |
| ESPRIT | Ethics committee (CCPPRB) of the Kremlin Bicetre hospital (n° registered 99-28) |
| H70-study | The study was approved by the Regional Ethical Review Board, and all methods were performed in accordance with the Helsinki Declaration. |
| HELIAD | Institutional Ethics Review Board of the University of Thessaly (ΒΕΥ846Ψ8Ν2-32Π) and of Aiginition Hospital (9Φ9Π46Ψ8Ν2-ΔΚΘ) |
| Invece.Ab | Ethics Committee of the University of Pavia (#3/2009) |
| ISA | UI/UCH Research Ethics Committee – UI/IRC/02/07P |
| Leiden85+ | Leiden University Medical Center, 1996 |
| LEILA75+ | Ethics committee of the University of Leipzig (C7 79934700) |
| LRGS TUA | National University of Malaysia (UKM) research ethics committee (reference: UKM PPI/111/8/JEP-2019-024), and was also approved by the Malaysian National Medical Research and Ethics Committee at the Malaysian Ministry of Health [reference: KKM/NIHSEC/P19-1689(12)] |
| MAAS | Ethics committee of Maastricht University Medical Centre (MEC05-107) |
| MYHAT | University of Pittsburgh Human Research Protection Office (formerly Institutional Review Board, IRB). Approval # PRO16030244 |
| SALSA | University of California, San Francisco Human Research Protection Program Institutional Review Board (IRB#10-00243) |
| SAS | Medical Ethics Committee of Huashan Hospital, Fudan University, Shanghai, China (approval number: HIRB2009-195) |
| SGS | Institutional Review Board of Fukuoka Institute of Technology (hm03-20-01) |
| SLAS II | National University of Singapore Institutional Review Board (Reference Code: 04-140) |
| SMAS | University of New South Wales Human Research Ethics Committee (approval #14327) |
| SPAH | Ethical Committee for the Analysis of Research Projects (CAPesq) - Hospital das Clínicas and Medical School - Project Registry Number: 257/2002; National Ethical Committee on Research (CONEP-Brazil) - Project Registry Number: 4355 |
| ZARADEMP | Ethics committee of the Zaragoza University Hospital (CEICA # CP16/2012) |

**Supplementary Table 16: Baseline characteristics for the 21 contributing COSMIC cohorts, by sex.**

|  | **BCSA** | | **CLAS** | | **EAS** | | **EPIDEMCA** | | **ESPRIT** | | **H-70 Study** | | **HELIAD** | |
| --- | --- | --- | --- | --- | --- | --- | --- | --- | --- | --- | --- | --- | --- | --- |
|  | **Women** | **Men** | **Women** | **Men** | **Women** | **Men** | **Women** | **Men** | **Women** | **Men** | **Women** | **Men** | **Women** | **Men** |
| Age (years) | 69.2 (7.2) | 68.7 (7.0) | 71.4 (7.8) | 71.3 (8.0) | 78.1 (5.4) | 77.7 (5.4) | 74.0 (6.8) | 73.0 (6.3) | 72.9 (5.5) | 72.9 (5.3) | 74.7 (5.3) | 70 (0.0) | 71.7 (5.2) | 73.9 (5.2) |
| Education (years) | 2.6 (2.9) | 3.0 (3.1) | 7.5 (5.3) | 9.8 (5.0) | 13.6 (3.5) | 13.8 (3.7) | 0.7 (2.0) | 4.5 (5.1) | 9.6 (3.0) | 10.7 (3.6) | 9.2 (3.0) | 11.4 (4.5) | 7.1 (4.5) | 8.5 (4.8) |
| Systolic blood pressure (mmHg) | 136.9 (22.3) | 137.9 (22.6) | 128.6 (14.7) | 129.6 (14.9) | 135.3 (16.1) | 134.1 (17.4) | 152.7 (31.1) | 143.9 (28.7) | 137.9 (16.5) | 145.0 (17.0) | 155.8 (22.6) | 156.7 (20.1) | 130.7 (17.1) | 132.5 (18.3) |
| Diastolic blood pressure (mmHg) | 82.4 (12.2) | 85.1 (13.1) | 76.9 (8.2) | 78.6 (9.2) | 77.9 (8.8) | 77.8 (8.5) | 84.0 (15.1) | 81.2 (16.0) | 78.1 (9.2) | 81.6 (10.1) | 83.8 (11.6) | 87.5 (9.7) | 76.8 (9.8) | 77.7 (10.2) |
| BMI (kg/m^2^) | 25.9 (5.3) | 23.9 (4.1) | 23.8 (3.5) | 23.3 (3.3) | 28.1 (5.8) | 28.3 (4.6) | 22.2 (5.6) | 21.3 (4.4) | 24.7 (3.9) | 25.7 (3.0) | 26.7 (4.2) | 26.9 (3.8) | 29.5 (4.7) | 28.5 (3.8) |
| Waist circumference (cm) | 92.1 (11.2) | 90.1 (11.1) | NA | NA | 92.2 (12.9) | 100.8 (10.6) | 82.3 (13.6) | 82.4 (12.2) | NA | NA | 87.5 (11.0) | 97.4 (11.1) | 98.5 (12.6) | 104.4 (10.4) |
| Hip circumference (cm) | 99.3 (10.6) | 96.5 (7.6) | NA | NA | 104.0 (11.9) | 104.2 (8.0) | 92.7 (11.9) | 90.5 (9.4) | NA | NA | 103.4 (10.8) | 102.0 (8.6) | NA | NA |
| Total cholesterol (mmol/l) | NA | NA | 5.2 (1.1) | 4.5 (1.0) | 5.1 (1.0) | 4.3 (0.9) | 4.4 (1.0) | 4.0 (0.9) | 6.0 (1.0) | 5.6 (0.9) | 6.1 (1.1) | 5.6 (1.0) | 5.6 (1.1) | 5.1 (1.1) |
| HDL cholesterol (mmol/l) | 1.3 (0.4) | 1.2 (0.4) | 1.3 (0.4) | 1.1 (0.3) | 1.6 (0.4) | 1.3 (0.3) | NA | NA | 1.7 (0.4) | 1.4 (0.3) | 1.7 (0.4) | 1.3 (0.4) | 1.6 (0.4) | 1.4 (0.3) |
| LDL cholesterol (mmol/l) | NA | NA | 3.1 (0.9) | 2.9 (2.0) | 2.9 (0.9) | 2.4 (0.8) | NA | NA | 3.7 (0.9) | 3.6 (0.8) | 3.8 (0.9) | 3.6 (0.9) | 3.3 (1.0) | 3.0 (0.9) |
| Triglycerides (mmol/l) (Median (Q1, Q3)) | 3.6 (2.6, 5.0) | 2.9 (2.1, 4.4) | 1.5 (1.1, 2.2) | 1.3 (0.9, 1.9) | 2.6 (1.9, 3.5) | 2.8 (1.9, 3.3) | NA | NA | 1.1 (0.9, 5.3) | 1.2 (0.9, 1.6) | NA | NA | 3.7 (2.7, 4.9) | 3.5 (2.6, 4.9) |
| Diabetes (N, %) | 137 (15.0) | 73 (12.5) | 192 (16.9) | 171 (17.7) | 118 (14.2) | 99 (19.0) | 108 (25.2) | 93 (30.2) | 59 (5.1) | 92 (11.3) | 78 (14.2) | 26 (14.4) | 125 (20.8) | 95 (23.4) |
| Depression (N, %) | 394 (43.2) | 165 (28.3) | 356 (31.4) | 236 (24.5) | 76 (8.9) | 40 (7.6) | 203 (47.3) | 88 (28.6) | 453 (39.0) | 177 (21.7) | 195 (35.4) | 37 (20.6) | 77 (12.8) | 25 (6.2) |
| Hearing impairment (N, %) | NA | NA | NA | NA | NA | NA | 8 (0.9) | 6 (1.1) | NA | NA | 311 (59.8) | 96 (54.5) | 80 (13.3) | 94 (23.2) |
| Current smoker (N, %) | 93 (10.2) | 178 (30.5) | 33 (2.9) | 272 (28.2) | 74 (8.9) | 38 (7.7) | 47 (11.0) | 51 (16.6) | 46 (4.0) | 79 (9.7) | 48 (8.7) | 25 (13.9) | 44 (7.3) | 62 (15.3) |
| Current alcohol use (N, %) | 46 (5.0) | 228 (39.0) | 39 (3.4) | 265 (27.5) | 575 (68.5) | 420 (80.0) | 29 (6.8) | 64 (20.8) | 892 (76.8) | 762 (93.5) | 358 (65.0) | 145 (80.6) | 156 (25.9) | 272 (67.0) |
| High physical activity (N, %) | 98 (10.7) | 105 (18.0) | 810 (71.5) | 721 (74.8) | 163 (19.4) | 128 (24.4) | 75 (17.5) | 68 (22.1) | 393 (33.8) | 289 (35.5) | 226 (41.0) | 159 (88.3) | 223 (37.0) | 201 (49.5) |
| APOE *ε*4 carrier (N, %) | 188 (20.6) | 127 (21.7) | NA | NA | 102 (12.1) | 56 (10.7) | 120 (28.0) | 74 (24.0) | 193 (16.6) | 158 (19.4) | 132 (24.0) | 51 (28.3) | 60 (10.0) | 60 (14.8) |
|  | **Invece.Ab** | | **ISA** | | **Leiden85+** | | **LEILA75+** | | **LRGS TUA** | | **MAAS** | | **MYHAT** | |
|  | **Women** | **Men** | **Women** | **Men** | **Women** | **Men** | **Women** | **Men** | **Women** | **Men** | **Women** | **Men** | **Women** | **Men** |
| Age (years) | 72.2 (1.3) | 62.1 (1.3) | 75.0 (8.7) | 73.1 (7.8) | 85.0 (0.0) | 85.0 (0.0) | 81.7 (4.8) | 81.2 (5.0) | 68.3 (6.0) | 69.1 (5.9) | 51.8 (16.2) | 51.3 (16.2) | 77.5 (7.4) | 77.3 (7.2) |
| Education (years) | 6.2 (2.9) | 7.6 (3.6) | 3.9 (5.1) | 4.3 (5.3) | 7.2 (2.8) | 8.1 (3.9) | 11.6 (1.8) | 12.8 (1.5) | 4.3 (3.9) | 6.3 (3.9) | 10.7 (3.0) | 11.7 (3.7) | 12.7 (2.2) | 13.3 (2.7) |
| Systolic blood pressure (mmHg) | 141.2 (17.5) | 142.7 (16.9) | NA | NA | 154.9 (18.1) | 156.3 (19.8) | 158.9 (24.5) | 157.6 (23.1) | 140.1 (22.2) | 141.6 (21.7) | 135.7 (22.7) | 139.9 (19.7) | 133.1 (15.4) | 133.6 (14.6) |
| Diastolic blood pressure (mmHg) | 78.8 (8.5) | 78.9 (8.2) | NA | NA | 77.6 (9.0) | 75.8 (10.0) | 86.3 (18.0) | 86.4 (16.2) | 76.9 (13.2) | 77.8 (13.4) | 72.5 (12.9) | 78.6 (11.7) | 73.9 (9.6) | 75.0 (9.0) |
| BMI (kg/m^2^) | 30.5 (6.6) | 25.4 (5.2) | NA | NA | 27.8 (4.7) | 26.3 (3.7) | NA | NA | 25.4 (4.6) | 24.6 (4.0) | 26.6 (4.6) | 26.9 (3.9) | 28.3 (6.0) | 27.9 (5.0) |
| Waist circumference (cm) | 95.6 (11.9) | 103.4 (10.5) | NA | NA | NA | NA | NA | NA | 87.1 (11.4) | 89.4 (10.9) | 84.4 (11.4) | 95.0 (10.2) | 91.4 (14.1) | 101.1 (12.1) |
| Hip circumference (cm) | NA | NA | NA | NA | NA | NA | NA | NA | 97.7 (10.0) | 95.4 (8.4) | 104.1 (9.4) | 104.1 (6.7) | 106.5 (13.4) | 105.2 (11.1) |
| Total cholesterol (mmol/l) | 5.9 (1.0) | 5.3 (0.9) | NA | NA | 5.9 (1.1) | 5.3 (0.9) | NA | NA | 3.8 (2.8) | 4.6 (3.1) | NA | NA | 5.3 (1.2) | 4.6 (1.0) |
| HDL cholesterol (mmol/l) | 1.5 (0.3) | 1.3 (0.3) | NA | NA | 1.4 (0.4) | 1.2 (0.3) | NA | NA | 1.5 (0.3) | 1.3 (0.3) | NA | NA | 1.3 (0.4) | 1.1 (0.3) |
| LDL cholesterol (mmol/l) | 3.7 (0.9) | 3.3 (0.8) | NA | NA | 3.8 (1.0) | 3.5 (0.8) | NA | NA | 3.4 (1.0) | 3.3 (1.0) | NA | NA | NA | NA |
| Triglycerides (mmol/l) (Median (Q1, Q3)) | 2.8 (2.2, 3.6) | 2.8 (2.1, 3.8) | NA | NA | 1.4 (1.1, 2.0) | 1.3 (1.0, 1.8) | NA | NA | 1.3 (1.0, 1.8) | 1.4 (1.0, 1.9) | NA | NA | NA | NA |
| Diabetes (N, %) | 110 (18.1) | 144 (27.7) | 10 (1.6) | 16 (2.6) | 141 (40.3) | 61 (36.1) | 165 (23.2) | 54 (21.6) | 270 (27.8) | 287 (30.8) | 31 (3.9) | 35 (4.2) | 463 (45.0) | 266 (42.6) |
| Depression (N, %) | 198 (32.6) | 64 (12.3) | 47 (7.4) | 38 (6.3) | 52 (14.9) | 25 (14.8) | 308 (43.3) | 69 (27.6) | 113 (11.6) | 66 (7.1) | 3 (0.4) | 0 (0.0) | 272 (26.4) | 110 (17.6) |
| Hearing impairment (N, %) | 80 (13.2) | 94 (18.1) | 14 (2.2) | 13 (2.1) | 93 (26.6) | 63 (37.3) | 204 (28.7) | 101 (40.4) | NA | NA | 57 (7.2) | 80 (9.5) | 214 (20.8) | 134 (21.4) |
| Current smoker (N, %) | 45 (7.4) | 55 (10.6) | NA | NA | 31 (8.9) | 45 (26.6) | 10 (1.4) | 6 (2.4) | 25 (2.6) | 296 (31.8) | 3 (0.4) | 12 (1.4) | 76 (7.4) | 31 (5.0) |
| Current alcohol use (N, %) | 304 (50.0) | 435 (83.8) | NA | NA | 152 (43.4) | 99 (58.6) | NA | NA | 15 (1.5) | 68 (7.3) | 581 (73.3) | 750 (89.1) | 625 (60.7) | 474 (75.8) |
| High physical activity (N, %) | 352 (57.9) | 382 (73.6) | 414 (64.8) | 448 (74.0) | NA | NA | NA | NA | 614 (63.2) | 836 (89.8) | 163 (20.6) | 272 (32.3) | 593 (57.6) | 401 (64.2) |
| APOE *ε*4 carrier (N, %) | 105 (17.3) | 87 (16.8) | NA | NA | 69 (19.7) | 31 (18.3) | 151 (21.2) | 41 (16.4) | NA | NA | 115 (14.5) | 123 (14.6) | 209 (20.3) | 111 (17.8) |
|  | **SALSA** | | **SAS** | | **SGS** | | **SLAS II** | | **SMAS** | | **SPAH** | | **ZARADEMP** | |
|  | **Women** | **Men** | **Women** | **Men** | **Women** | **Men** | **Women** | **Men** | **Women** | **Men** | **Women** | **Men** | **Women** | **Men** |
| Age (years) | 70.2 (6.8) | 70.1 (6.6) | 71.6 (7.5) | 71.4 (7.3) | 73.0 (5.9) | 72.4 (5.3) | 65.0 (6.8) | 66.2 (7.0) | 78.6 (4.9) | 78.6 (4.6) | 71.8 (5.8) | 71.3 (5.6) | 72.2 (8.7) | 71.7 (8.8) |
| Education (years) | 7.1 (5.1) | 7.9 (5.6) | 10.9 (4.3) | 13.1 (3.4) | 10.9 (2.1) | 12.0 (3.0) | 5.8 (4.5) | 7.9 (4.3) | 11.1 (3.0) | 12.3 (3.8) | 2.3 (2.8) | 3.2 (3.5) | 7.0 (3.5) | 8.2 (4.2) |
| Systolic blood pressure (mmHg) | 137.3 (19.3) | 140.1 (19.1) | 144.4 (23.1) | 147.8 (22.3) | 149.9 (21.4) | 151.4 (21.4) | 126.4 (14.4) | 127.7 (14.8) | 143.2 (21.4) | 147.3 (20.8) | 143.4 (24.5) | 147.5 (26.9) | 141.5 (18.5) | 140.5 (19.1) |
| Diastolic blood pressure (mmHg) | 74.5 (10.3) | 78.0 (10.5) | 75.6 (11.4) | 79.3 (11.4) | 80.4 (11.8) | 82.5 (11.4) | 79.5 (8.9) | 81.4 (9.7) | 81.7 (10.9) | 81.8 (11.9) | 84.7 (12.9) | 87.7 (13.7) | 79.9 (11.1) | 78.6 (11.0) |
| BMI (kg/m^2^) | 30.1 (6.1) | 29.3 (5.0) | 24.5 (3.6) | 24.8 (3.3) | 23.2 (3.2) | 23.2 (2.6) | 24.1 (4.1) | 23.9 (3.6) | 26.7 (4.6) | 27.6 (4.2) | 26.8 (5.0) | 25.0 (3.8) | 27.2 (6.1) | 26.8 (4.7) |
| Waist circumference (cm) | 94.9 (13.6) | 99.8 (12.1) | NA | NA | NA | NA | 82.2 (10.6) | 88.6 (9.6) | 91.1 (11.9) | 102.8 (10.9) | NA | NA | NA | NA |
| Hip circumference (cm) | NA | NA | NA | NA | NA | NA | 96.8 (9.1) | 95.9 (7.9) | 106.2 (10.5) | 107.5 (9.1) | NA | NA | NA | NA |
| Total cholesterol (mmol/l) | 5.6 (1.0) | 5.3 (1.0) | 5.7 (1.0) | 5.1 (1.0) | NA | NA | 5.4 (1.0) | 5.0 (1.0) | 5.0 (1.0) | 4.5 (0.9) | 5.7 (1.1) | 5.1 (1.1) | NA | NA |
| HDL cholesterol (mmol/l) | 1.4 (0.4) | 1.2 (0.3) | 1.4 (0.4) | 1.2 (0.3) | NA | NA | 1.6 (0.4) | 1.3 (0.3) | 1.6 (0.4) | 1.3 (0.4) | 1.5 (0.4) | 1.3 (0.4) | NA | NA |
| LDL cholesterol (mmol/l) | 3.2 (0.9) | 3.2 (0.9) | 3.5 (0.9) | 3.2 (0.9) | NA | NA | 3.2 (0.9) | 3.1 (0.9) | 2.9 (0.9) | 2.7 (0.8) | 3.4 (0.9) | 3.1 (0.8) | NA | NA |
| Triglycerides (mmol/l) (Median (Q1, Q3)) | 4.2 (3.2, 5.9) | 3.9 (2.6, 5.8) | 1.5 (1.2, 2.1) | 1.4 (1.0, 2.0) | NA | NA | 1.1 (0.8, 1.5) | 1.2 (0.9, 1.6) | 1.0 (0.7, 1.3) | (0.9, 0.7, 1.3) | 3.3 (2.4, 4.9) | 3.2 (2.2, 4.5) | NA | NA |
| Diabetes (N, %) | 283 (30.1) | 226 (33.2) | 154 (17.1) | 138 (18.2) | 46 (8.8) | 70 (17.0) | 136 (14.6) | 86 (17.0) | 49 (9.9) | 88 (21.3) | 215 (21.8) | 119 (19.4) | 217 (12.1) | 177 (12.2) |
| Depression (N, %) | 351 (37.3) | 130 (19.1) | 542 (60.2) | 446 (58.8) | 31 (6.0) | 24 (5.8) | 35 (3.7) | 14 (2.8) | 119 (24.1) | 92 (22.2) | 98 (10.0) | 31 (5.0) | 430 (24.0) | 116 (8.0) |
| Hearing impairment (N, %) | NA | NA | NA | NA | NA | NA | NA | NA | 180 (36.5) | 187 (45.2) | 13 (1.3) | 9 (1.5) | NA | NA |
| Current smoker (N, %) | 12 (1.3) | 18 (2.6) | 8 (0.9) | 161 (21.2) | 16 (3.1) | 58 (14.1) | 18 (1.9) | 97 (19.2) | 20 (4.1) | 12 (2.9) | 100 (10.2) | 98 (16.0) | 57 (3.2) | 396 (27.3) |
| Current alcohol use (N, %) | 432 (45.9) | 459 (67.5) | 15 (1.7) | 134 (17.7) | 139 (26.7) | 281 (68.7) | 32 (3.4) | 85 (16.8) | 450 (91.3) | 401 (96.9) | 541 (55.0) | 532 (86.6) | 295 (16.5) | 983 (67.9) |
| High physical activity (N, %) | NA | NA | 177 (19.7) | 235 (31.0) | 357 (68.5) | 251 (61.1) | 901 (96.5) | 492 (97.2) | 431 (87.4) | 374 (90.3) | 800 (81.3) | 494 (80.5) | NA | NA |
| APOE *ε*4 carrier (N, %) | NA | NA | NA | NA | NA | NA | 147 (15.7) | 68 (13.4) | 98 (19.9) | 90 (21.7) | 184 (18.7) | 118 (19.2) | NA | NA |

Presented as mean (SD) unless stated otherwise.

**Supplementary Table 17: APOE *ε*4 carriage (versus *ε*3/*ε*3) and the association with dementia by sex, by age and region subgroups.**

|  | **Women**  **HR (95% CI)** | **Men**  **HR (95% CI)** | **Women-to-men**  **RHR (95% CI)** | **P for interaction** |
| --- | --- | --- | --- | --- |
| Baseline age: |  |  |  |  |
| <75 years | 1.55 (1.18, 2.04) | 2.14 (1.73, 2.66) | 0.73 (0.51, 1.03) | 0.27 |
| ≥75 years | 1.69 (1.37, 2.10) | 1.74 (1.49, 2.03) | 0.97 (0.76, 1.25) |  |
| Region: |  |  |  |  |
| Western countries | 1.73 (1.38, 2.16) | 2.21 (1.88, 2.61) | 0.78 (0.60, 1.02) | 0.25 |
| Asian countries | 1.52 (0.95, 2.42) | 1.63 (1.15, 2.30) | 0.93 (0.52, 1.66) |  |
| Other | 1.51 (0.97, 2.36) | 1.35 (0.99, 1.84) | 1.12 (0.65, 1.93) |  |

**Supplementary Table 18: Age- and education-adjusted hazard ratios for risk factors associated with the risk of incident all-cause dementia by sex, and women-to-men ratio of hazard ratios.**

|  | **complete case** | | | **MICE** | | |
| --- | --- | --- | --- | --- | --- | --- |
| **Risk factor** | **Women**  **HR (95% CI)** | **Men**  **HR (95% CI)** | **Women-to-men**  **RHR (95% CI)** | **Women**  **HR (95% CI)** | **Men**  **HR (95% CI)** | **Women-to-men**  **RHR (95% CI)** |
| Age (per year) | 1.11 (1.10, 1.11) | 1.11 (1.10, 1.12) | 1.00 (0.99, 1.01) | 1.11 (1.10, 1.12) | 1.11 (1.10, 1.12) | 1.00 (0.99, 1.01) |
| Education (per year) | 0.95 (0.93, 0.97) | 0.94 (0.92, 0.95) | 1.01 (1.00, 1.03) | 0.95 (0.93, 0.97) | 0.94 (0.92, 0.95) | 1.01 (1.00, 1.03) |
| Systolic blood pressure (per 20 mmHg) | 1.03 (0.95, 1.11) | 0.96 (0.91, 1.02) | 1.07 (0.97, 1.17) | 1.02 (0.95, 1.10) | 0.96 (0.92, 1.02) | 1.06 (0.98, 1.15) |
| Diastolic blood pressure (per 10 mmHg) | 1.02 (0.95, 1.09) | 0.99 (0.95, 1.04) | 1.02 (0.94, 1.11) | 0.99 (0.93, 1.06) | 1.00 (0.95, 1.04) | 1.00 (0.92, 1.07) |
| Hypertension (vs no) | 1.01 (0.84, 1.21) | 1.01 (0.87, 1.16) | 1.00 (0.81, 1.23) | 0.98 (0.83, 1.17) | 1.00 (0.87, 1.14) | 0.98 (0.81, 1.20) |
| Body mass index (per 5 kg/m^2^) | 1.00 (0.91, 1.10) | 0.89 (0.84, 0.96) | 1.12 (1.00, 1.25) | 0.97 (0.89, 1.05) | 0.88 (0.83, 0.93) | 1.10 (0.99, 1.22) |
| Waist circumference (per 10 cm) | 1.03 (0.93, 1.13) | 0.95 (0.88, 1.01) | 1.09 (0.97, 1.22) | 1.01 (0.94, 1.08) | 0.94 (0.90, 0.99) | 1.07 (0.99, 1.16) |
| Hip circumference (per 5 cm) | 0.94 (0.87, 1.02) | 0.94 (0.90, 0.99) | 1.01 (0.92, 1.10) | 0.96 (0.92, 0.99) | 0.94 (0.91, 0.96) | 1.02 (0.97, 1.06) |
| Total cholesterol (per mmol/l) | 0.93 (0.86, 1.02) | 1.00 (0.94, 1.06) | 0.93 (0.84, 1.04) | 0.96 (0.91, 1.02) | 1.01 (0.97, 1.05) | 0.96 (0.90, 1.02) |
| HDL cholesterol (per mmol/l) | 0.75 (0.56, 1.01) | 0.90 (0.75, 1.09) | 0.84 (0.59, 1.18) | 0.83 (0.66, 1.05) | 0.88 (0.77, 1.02) | 0.94 (0.72, 1.23) |
| LDL cholesterol (per mmol/l) | 0.92 (0.81, 1.03) | 1.08 (0.99, 1.18) | 0.85 (0.74, 0.98) | 1.02 (0.95, 1.10) | 1.07 (1.02, 1.13) | 0.96 (0.88, 1.04) |
| Triglycerides (per mmol/l) | 1.02 (0.96, 1.08) | 1.03 (0.99, 1.08) | 0.99 (0.93, 1.06) | 1.01 (0.97, 1.05) | 1.03 (1.00, 1.05) | 0.98 (0.94, 1.03) |
| High cholesterol (vs no) | 0.87 (0.70, 1.08) | 1.01 (0.87, 1.17) | 0.86 (0.67, 1.12) | 0.92 (0.75, 1.13) | 0.94 (0.82, 1.07) | 0.98 (0.77, 1.23) |
| Diabetes (vs no) | 1.18 (0.98, 1.42) | 1.19 (1.04, 1.36) | 0.99 (0.79, 1.25) | 1.18 (1.00, 1.42) | 1.19 (1.04, 1.37) | 0.99 (0.79, 1.24) |
| Depression (vs no) | 1.37 (1.14, 1.64) | 1.32 (1.17, 1.48) | 1.04 (0.84, 1.28) | 1.39 (1.16, 1.65) | 1.30 (1.16, 1.46) | 1.06 (0.87, 1.30) |
| Hearing impairment (vs no) | 1.05 (0.82, 1.35) | 1.24 (1.04, 1.49) | 0.85 (0.62, 1.15) | 1.17 (1.00, 1.38) | 1.18 (1.04, 1.34) | 0.99 (0.80, 1.20) |
| Current smoker (vs never) | 0.98 (0.73, 1.31) | 0.86 (0.63, 1.17) | 1.14 (0.76, 1.72) | 0.96 (0.76, 1.21) | 0.81 (0.64, 1.03) | 1.18 (0.85, 1.63) |
| Former smoker (vs never) | 0.97 (0.81, 1.17) | 0.91 (0.76, 1.10) | 1.06 (0.83, 1.36) | 0.94 (0.80, 1.11) | 0.91 (0.77, 1.07) | 1.04 (0.83, 1.31) |
| Current alcohol use (vs never) | 0.71 (0.53, 0.95) | 0.77 (0.62, 0.95) | 0.93 (0.68, 1.26) | 0.69 (0.51, 0.93) | 0.80 (0.65, 0.96) | 0.87 (0.64, 1.18) |
| Former alcohol use (vs never) | 0.78 (0.57, 1.08) | 0.96 (0.77, 1.20) | 0.82 (0.60, 1.11) | 0.65 (0.43, 0.98) | 1.18 (0.88, 1.59) | 0.55 (0.38, 0.79) |
| High physical activity (vs none to minimal) | 0.69 (0.58, 0.83) | 0.73 (0.63, 0.86) | 0.94 (0.75, 1.18) | 0.69 (0.59, 0.81) | 0.72 (0.63, 0.82) | 0.95 (0.79, 1.15) |
| APOE *ε*2 carriage (vs *ε*3/*ε3*) | 1.01 (0.72, 1.41) | 0.99 (0.71, 1.38) | 1.02 (0.68, 1.54) | 1.00 (0.78, 1.27) | 0.98 (0.83, 1.18) | 1.01 (0.75, 1.36) |
| APOE *ε*4 carriage (vs *ε*3/*ε*3) | 1.67 (1.31, 2.11) | 2.09 (1.65, 2.65) | 0.80 (0.60, 1.06) | 1.63 (1.38, 1.93) | 1.87 (1.65, 2.12) | 0.88 (0.71, 1.07) |

HR, hazard ratio; CI, confidence interval; HDL, high density lipoprotein; LDL, low density lipoprotein; APOE, apolipoprotein E; RHR, ratio of hazard ratios; MICE, Multiple Imputation by Chained Equations.

**Supplementary Table 19: Age- and education-adjusted and multiple-adjusted hazard ratios for risk factors associated with the risk of incident all-cause dementia by sex, and women-to-men ratio of the hazard ratios, using imputed dataset.**

| **Risk factor** | **Age- and education-adjusted** | | | **Multiple-adjusted*** | | |
| --- | --- | --- | --- | --- | --- | --- |
| Sex (women versus men) | 1.12 (1.03, 1.23) | | | 1.10 (1.00, 1.20) | | |
|  | **Women**  **HR (95% CI)** | **Men**  **HR (95% CI)** | **Women-to-men**  **RHR (95% CI)** | **Women**  **HR (95% CI)** | **Men**  **HR (95% CI)** | **Women-to-men**  **RHR (95% CI)** |
| Age (per year) | 1.11 (1.10, 1.12) | 1.11 (1.10, 1.12) | 1.00 (0.99, 1.01) | 1.11 (1.10, 1.12) | 1.11 (1.10, 1.12) | 1.00 (0.99, 1.01) |
| Education (per year) | 0.95 (0.93, 0.97) | 0.94 (0.92, 0.95) | 1.01 (1.00, 1.03) | 0.95 (0.93, 0.96) | 0.94 (0.92, 0.95) | 1.01 (0.99, 1.03) |
| Systolic blood pressure (per 20 mmHg) | 1.02 (0.95, 1.10) | 0.96 (0.92, 1.02) | 1.06 (0.98, 1.15) | 1.05 (0.97, 1.12) | 0.97 (0.92, 1.02) | 1.08 (0.99, 1.18) |
| Diastolic blood pressure (per 10 mmHg) | 0.99 (0.93, 1.06) | 1.00 (0.95, 1.04) | 1.00 (0.92, 1.07) | 1.00 (0.95, 1.08) | 1.02 (0.97, 1.06) | 0.99 (0.92, 1.07) |
| Hypertension (vs no) | 0.98 (0.83, 1.17) | 1.00 (0.87, 1.14) | 0.98 (0.81, 1.20) | 0.98 (0.83, 1.17) | 1.03 (0.90, 1.18) | 0.95 (0.78, 1.17) |
| Body mass index (per 5 kg/m^2^) | 0.97 (0.89, 1.05) | 0.88 (0.83, 0.93) | 1.10 (0.99, 1.22) | 0.97 (0.89, 1.06) | 0.88 (0.83, 0.93) | 1.10 (0.99, 1.22) |
| Waist circumference (per 10 cm) | 1.01 (0.94, 1.08) | 0.94 (0.90, 0.99) | 1.07 (0.99, 1.16) | 1.00 (0.93, 1.07) | 0.94 (0.89, 0.98) | 1.07 (0.98, 1.16) |
| Hip circumference (per 5 cm) | 0.96 (0.92, 0.99) | 0.94 (0.91, 0.96) | 1.02 (0.97, 1.06) | 0.95 (0.91, 0.99) | 0.94 (0.92, 0.97) | 1.01 (0.97, 1.06) |
| Total cholesterol (per mmol/l) | 0.96 (0.91, 1.02) | 1.01 (0.97, 1.05) | 0.96 (0.90, 1.02) | 0.95 (0.89, 1.00) | 0.98 (0.94, 1.02) | 0.97 (0.91, 1.04) |
| HDL cholesterol (per mmol/l) | 0.83 (0.66, 1.05) | 0.88 (0.77, 1.02) | 0.94 (0.72, 1.23) | 0.83 (0.65, 1.05) | 0.86 (0.74, 1.00) | 0.96 (0.73, 1.26) |
| LDL cholesterol (per mmol/l) | 1.02 (0.95, 1.10) | 1.07 (1.02, 1.13) | 0.96 (0.88, 1.04) | 1.01 (0.93, 1.09) | 1.04 (0.99, 1.10) | 0.96 (0.88, 1.06) |
| Triglycerides (per mmol/l) | 1.01 (0.97, 1.05) | 1.03 (1.00, 1.05) | 0.98 (0.94, 1.03) | 1.00 (0.96, 1.05) | 1.03 (1,00, 1.06) | 0.98 (0.93, 1.03) |
| High cholesterol (vs no) | 0.92 (0.75, 1.13) | 0.94 (0.82, 1.07) | 0.98 (0.77, 1.23) | 0.88 (0.72, 1.08) | 0.91 (0.80, 1.04) | 0.97 (0.76, 1.23) |
| Diabetes (vs no) | 1.18 (1.00, 1.42) | 1.19 (1.04, 1.37) | 0.99 (0.79, 1.24) | 1.17 (0.97, 1.41) | 1.26 (1.10, 1.45) | 0.92 (0.74, 1.16) |
| Depression (vs no) | 1.39 (1.16, 1.65) | 1.30 (1.16, 1.46) | 1.06 (0.87, 1.30) | 1.40 (1.17, 1.67) | 1.29 (1.15, 1.45) | 1.08 (0.88, 1.33) |
| Hearing impairment (vs no) | 1.17 (1.00, 1.38) | 1.18 (1.04, 1.34) | 0.99 (0.80, 1.20) | 1.12 (0.95, 1.33) | 1.19 (1.05, 1.35) | 0.94 (0.77, 1.15) |
| Current smoker (vs never) | 0.96 (0.76, 1.21) | 0.81 (0.64, 1.03) | 1.18 (0.85, 1.63) | 0.95 (0.75, 1.20) | 0.77 (0.50, 0.98) | 1.23 (0.89, 1.72) |
| Former smoker (vs never) | 0.94 (0.80, 1.11) | 0.91 (0.77, 1.07) | 1.04 (0.83, 1.31) | 0.93 (0.79, 1.10) | 0.89 (0.84, 1.32) | 1.05 (0.84, 1.32) |
| Current alcohol use (vs never) | 0.69 (0.51, 0.93) | 0.80 (0.65, 0.96) | 0.87 (0.64, 1.18) | 0.68 (0.51, 0.92) | 0.81 (0.66, 0.98) | 0.85 (0.62, 1.15) |
| Former alcohol use (vs never) | 0.65 (0.43, 0.98) | 1.18 (0.88, 1.59) | 0.55 (0.38, 0.79) | 0.65 (0.43, 0.99) | 1.16 (0.86, 1.56) | 0.56 (0.38, 0.82) |
| High physical activity (vs none to minimal) | 0.69 (0.59, 0.81) | 0.72 (0.63, 0.82) | 0.95 (0.79, 1.15) | 0.68 (0.58, 0.80) | 0.72 (0.63, 0.82) | 0.94 (0.78, 1.15) |
| APOE *ε*2 carriage (vs *ε*3/*ε3*) | 1.00 (0.78, 1.27) | 0.98 (0.83, 1.18) | 1.01 (0.75, 1.36) | 1.01 (0.79, 1.28) | 0.99 (0.83, 1.18) | 1.02 (0.75, 1.37) |
| APOE *ε*4 carriage (vs *ε*3/*ε*3) | 1.63 (1.38, 1.93) | 1.87 (1.65, 2.12) | 0.88 (0.71, 1.07) | 1.66 (1.40, 1.97) | 1.86 (1.64, 2.11) | 0.89 (0.73, 1.10) |

HR, hazard ratio; CI, confidence interval; BMI, body mass index; HDL, high density lipoprotein; LDL, low density lipoprotein; APOE, apolipoprotein E; RHR, ratio of hazard ratios.

* In addition to adjusting for age and education years, age and education were adjusted for systolic blood pressure, BMI, total cholesterol, diabetes, depression, smoking status, and APOE genotype; blood pressure indices (systolic and diastolic blood pressure) were adjusted for BMI, total cholesterol, diabetes, depression, smoking status, and APOE genotype; body anthropometry indices (BMI, waist and hip circumference) were adjusted for systolic blood pressure, total cholesterol, diabetes, depression, smoking status, and APOE genotype; lipid indices (total, HDL, LDL cholesterol, triglycerides) were adjusted for systolic blood pressure, BMI, diabetes, depression, smoking status, and APOE genotype; pre-existing health conditions (diabetes, depression and hearing impairment) were adjusted for systolic blood pressure, BMI, total cholesterol, diabetes, depression, smoking status, and APOE genotype; lifestyle factors (smoking, alcohol use and physical activity) were adjusted for systolic blood pressure, BMI, total cholesterol, diabetes, depression, smoking status, and APOE genotype; and APOE *ε*4 status was adjusted for systolic blood pressure, BMI, total cholesterol, diabetes, depression, smoking status.

**Supplementary Table 20: Age- and education-adjusted women-to-men ratio of hazard ratios for all-cause dementia by risk factor, in cohorts used DSM for dementia diagnosis only.**

| **Risk factor** | **Age- and education-adjusted** | | |
| --- | --- | --- | --- |
| Sex (women versus men) | 1.02 (0.91, 1.15) | | |
|  | **Women**  **HR (95% CI)** | **Men**  **HR (95% CI)** | **Women-to-men**  **RHR (95% CI)** |
| Age (per year) | 1.13 (1.11, 1.14) | 1.13 (1.12, 1.14) | 0.99 (0.98, 1.01) |
| Education (per year) | 0.95 (0.93, 0.97) | 0.95 (0.93, 0.97) | 1.00 (0.98, 1.03) |
| Systolic blood pressure (per 20 mmHg) | 1.02 (0.93, 1.11) | 0.97 (0.91, 1.04) | 1.04 (0.94, 1.16) |
| Diastolic blood pressure (per 10 mmHg) | 1.02 (0.95, 1.11) | 0.98 (0.93, 1.03) | 1.05 (0.95, 1.15) |
| Hypertension (vs no) | 1.01 (0.81, 1.27) | 0.93 (0.78, 1.12) | 1.08 (0.85, 1.38) |
| Body mass index (per 5 kg/m^2^) | 0.99 (0.89, 1.09) | 0.89 (0.83, 0.96) | 1.11 (0.98, 1.25) |
| Waist circumference (per 10 cm) | 1.05 (0.97, 1.14) | 0.94 (0.89, 1.00) | 1.11 (1.01, 1.22) |
| Hip circumference (per 5 cm) | 0.98 (0.93, 1.03) | 0.94 (0.91, 0.98) | 1.03 (0.98, 1.09) |
| Total cholesterol (per mmol/l) | 0.98 (0.91, 1.07) | 1.01 (0.96, 1.07) | 0.97 (0.88, 1.07) |
| HDL cholesterol (per mmol/l) | 0.75 (0.56, 1.00) | 0.86 (0.72, 1.02) | 0.87 (0.63, 1.22) |
| LDL cholesterol (per mmol/l) | 1.00 (0.90, 1.11) | 1.08 (1.00, 1.16) | 0.93 (0.82, 1.05) |
| Triglycerides (per mmol/l) | 1.01 (0.96, 1.06) | 1.03 (1.00, 1.06) | 0.98 (0.93, 1.03) |
| High cholesterol (vs no) | 1.07 (0.84, 1.36) | 1.07 (0.91, 1.26) | 1.00 (0.76, 1.32) |
| Diabetes (vs no) | 1.32 (1.06, 1.65) | 1.44 (1.22, 1.71) | 0.92 (0.69, 1.21) |
| Depression (vs no) | 1.58 (1.26, 1.97) | 1.47 (1.27, 1.69) | 1.08 (0.83, 1.39) |
| Hearing impairment (vs no) | 0.99 (0.81, 1.22) | 1.13 (0.97, 1.32) | 0.87 (0.68, 1.12) |
| Current smoker (vs never) | 0.88 (0.61, 1.27) | 0.82 (0.57, 1.17) | 1.07 (0.65, 1.79) |
| Former smoker (vs never) | 1.00 (0.82, 1.23) | 0.94 (0.76, 1.15) | 1.08 (0.81, 1.41) |
| Current alcohol use (vs never) | 0.83 (0.60, 1.17) | 0.84 (0.66, 1.06) | 1.00 (0.70, 1.43) |
| Former alcohol use (vs never) | 0.93 (0.63, 1.39) | 1.01 (0.78, 1.32) | 0.92 (0.62, 1.37) |
| High physical activity (vs none to minimal) | 0.71 (0.58, 0.87) | 0.66 (0.56, 0.79) | 1.08 (0.85, 1.37) |
| *APOE ε2* carriage (vs *ε*3/*ε3*) | 1.05 (0.79, 1.29) | 1.04 (0.84, 1.29) | 1.01 (0.70, 1.45) |
| *APOE ε4* carriage (vs *ε*3/*ε*3) | 1.72 (1.40, 2.11) | 2.07 (1.76, 2.43) | 0.83 (0.65, 1.07) |

**Supplementary Table 21: Missing data patterns.**

| **Participants %** | **Participants N** | **Education years** | **Age** | **diabetes** | **depression** | **Alcohol use** | **Systolic blood pressure** | **Diastolic blood pressure** | **Smoking** | **Body mass index** | **Physical activity** | **HDL cholesterol** | **Total cholesterol** | **Waist circumference** | **LDL cholesterol** | **Triglycerides** | **APOE 4** | **Hearing impairment** | **Hip circumference** | **Number of missing variables** |
| --- | --- | --- | --- | --- | --- | --- | --- | --- | --- | --- | --- | --- | --- | --- | --- | --- | --- | --- | --- | --- |
| *9.65* | *2881* | *1* | *1* | *1* | *1* | *1* | *1* | *1* | *1* | *1* | *0* | *0* | *0* | *0* | *0* | *0* | *0* | *0* | *0* | *9* |
| *6.84* | *2042* | *1* | *1* | *1* | *1* | *1* | *1* | *1* | *1* | *1* | *1* | *1* | *1* | *0* | *1* | *1* | *0* | *0* | *0* | *4* |
| *5.34* | *1593* | *1* | *1* | *1* | *1* | *1* | *1* | *1* | *1* | *1* | *1* | *1* | *1* | *0* | *1* | *1* | *1* | *0* | *0* | *3* |
| *5.04* | *1505* | *1* | *1* | *1* | *1* | *1* | *1* | *1* | *1* | *1* | *1* | *1* | *1* | *1* | *1* | *1* | *0* | *0* | *1* | *2* |
| 4.98 | 1488 | 1 | 1 | 1 | 1 | 1 | 1 | 1 | 1 | 1 | 1 | 0 | 0 | 0 | 0 | 0 | 0 | 0 | 0 | 8 |
| 4.72 | 1408 | 1 | 1 | 1 | 1 | 1 | 1 | 1 | 1 | 1 | 1 | 1 | 1 | 1 | 1 | 1 | 1 | 1 | 0 | 1 |
| 4.69 | 1399 | 1 | 1 | 1 | 1 | 1 | 1 | 1 | 1 | 1 | 1 | 1 | 1 | 1 | 1 | 1 | 1 | 0 | 1 | 1 |
| 4.31 | 1286 | 1 | 1 | 1 | 1 | 1 | 1 | 1 | 1 | 1 | 1 | 1 | 0 | 1 | 0 | 1 | 1 | 0 | 1 | 3 |
| 3.97 | 1186 | 1 | 1 | 1 | 1 | 1 | 1 | 1 | 1 | 1 | 0 | 1 | 1 | 1 | 1 | 1 | 0 | 0 | 0 | 4 |
| 3.89 | 1161 | 1 | 1 | 1 | 1 | 0 | 0 | 0 | 0 | 0 | 1 | 0 | 0 | 0 | 0 | 0 | 0 | 1 | 0 | 12 |
| 3.32 | 991 | 1 | 1 | 1 | 1 | 1 | 1 | 1 | 1 | 1 | 1 | 0 | 0 | 1 | 0 | 0 | 1 | 1 | 1 | 4 |
| 2.89 | 863 | 1 | 1 | 1 | 1 | 1 | 1 | 1 | 1 | 1 | 1 | 1 | 1 | 0 | 1 | 0 | 1 | 1 | 0 | 3 |
| *2.61* | *779* | *1* | *1* | *1* | *1* | *1* | *1* | *1* | *1* | *1* | *1* | *1* | *1* | *1* | *1* | *1* | *1* | *1* | *1* | *0* |
| 2.04 | 610 | 1 | 1 | 1 | 1 | 1 | 1 | 1 | 1 | 1 | 1 | 0 | 0 | 1 | 0 | 0 | 0 | 1 | 1 | 5 |
| 2.00 | 597 | 1 | 1 | 1 | 1 | 0 | 1 | 1 | 1 | 0 | 0 | 0 | 0 | 0 | 0 | 0 | 0 | 1 | 0 | 10 |
| 1.66 | 495 | 1 | 1 | 1 | 1 | 1 | 1 | 1 | 0 | 1 | 1 | 0 | 0 | 1 | 0 | 0 | 1 | 1 | 1 | 5 |
| 1.55 | 462 | 1 | 1 | 1 | 1 | 1 | 1 | 1 | 1 | 1 | 1 | 0 | 1 | 1 | 0 | 0 | 1 | 1 | 1 | 3 |
| 1.53 | 456 | 1 | 1 | 1 | 1 | 1 | 1 | 1 | 1 | 1 | 1 | 1 | 1 | 0 | 1 | 1 | 1 | 1 | 0 | 2 |
| 1.49 | 446 | 1 | 1 | 1 | 1 | 1 | 1 | 1 | 1 | 1 | 1 | 1 | 1 | 1 | 0 | 0 | 1 | 1 | 1 | 2 |
| 1.38 | 412 | 1 | 1 | 1 | 1 | 1 | 1 | 1 | 1 | 1 | 0 | 1 | 1 | 0 | 1 | 1 | 1 | 1 | 0 | 3 |
| 1.32 | 393 | 1 | 1 | 1 | 1 | 1 | 1 | 1 | 0 | 1 | 1 | 1 | 1 | 1 | 0 | 0 | 1 | 1 | 1 | 3 |
| 1.13 | 338 | 1 | 1 | 1 | 1 | 1 | 1 | 1 | 1 | 1 | 1 | 1 | 1 | 1 | 1 | 0 | 1 | 1 | 1 | 1 |
| 1.13 | 337 | 1 | 1 | 1 | 1 | 1 | 1 | 1 | 1 | 0 | 1 | 0 | 0 | 0 | 0 | 0 | 0 | 0 | 0 | 9 |
| 1.07 | 319 | 1 | 1 | 1 | 1 | 1 | 1 | 1 | 0 | 1 | 0 | 1 | 1 | 1 | 1 | 1 | 0 | 0 | 0 | 5 |
| 1.06 | 315 | 1 | 1 | 1 | 1 | 1 | 1 | 1 | 1 | 1 | 1 | 0 | 1 | 1 | 0 | 0 | 0 | 0 | 1 | 5 |
| **Number missing from each variable** |  | 112 | 249 | 390 | 619 | 2374 | 3216 | 3264 | 3699 | 4419 | 8094 | 13149 | 13549 | 15509 | 15521 | 15596 | 15907 | 17860 | 19116 | **Total number missing**  152643 |
| **% Missing from each variable** |  | 0.38 | 0.83 | 1.31 | 2.07 | 7.95 | 10.77 | 10.93 | 12.39 | 14.80 | 27.12 | 44.05 | 45.39 | 51.96 | 52.00 | 52.25 | 53.29 | 59.83 | 64.04 |  |

Note: In the table, 1 represents complete variables and 0 represents missing variables
